# Effect of age and sex on the urinary elimination of a single dose of mixed flavonoids: results from a single-arm intervention in healthy United Kingdom adults

**DOI:** 10.1016/j.ajcnut.2025.05.006

**Published:** 2025-05-12

**Authors:** Colin D Kay, Noemi Tejera, Amy Jennings, Sumanto Haldar, Bethany C. Diment, Damon Bevan, Lisa C Crossman, Sherly Li, Aedin Cassidy, Anne-Marie Minihane

**Affiliations:** 1 Department of Paediatrics, Arkansas Children’s Research Institute - Arkansas Children’s Nutrition Center, Little Rock, AR, United States; 2 Department of Nutrition and Preventive Medicine, Norwich Medical School, University of East Anglia (UEA), Norwich, United Kingdom; 3 Institute for Global Food Security, Queen's University Belfast, Belfast, Northern Ireland; 4 Faculty of Health and Social Sciences, Bournemouth University, Bournemouth, United Kingdom; 5 School of Biological Sciences, University of East Anglia (UEA), Norwich, United Kingdom; 6 SequenceAnalysis.co.uk, NRP Innovation Centre, Norwich Research Park, Norwich, United Kingdom; 7 Murdoch Children’s Research Institute, Melbourne, Australia; 8 Medical Research Council Epidemiology Unit, University of Cambridge, Cambridge, United Kingdom; 9 Norwich Institute of Healthy Ageing, Norwich, United Kingdom

**Keywords:** absorption, metabolism, excretion, flavonoids, polyphenols, genes, gut microflora

## Abstract

**Background:**

Nutrition intervention trials demonstrate that increased flavonoid intake can have clinically meaningful impacts on disease outcomes/biomarkers; however, high variability in absorption and metabolism and large heterogeneity in biochemical and physiological responses are observed. The etiology of this variability is poorly understood.

**Objective:**

The objective of this study was to explore the relationships between sex, age, and microbiota speciation on mixed flavonoid elimination over 24 h.

**Methods:**

Healthy males and females (*n* = 163) prospectively recruited on the basis of age (18–30 y or 65–77 y) and sex consumed a standardized flavonoid-rich test meal providing 640-mg cocoa/chocolate flavan-3-ols, 340-mg citrus flavanones, and 390-mg blackberry anthocyanins. Urinary samples collected at baseline (−24 to 0 h), 0 to 3.5 h, >3.5 h to 7 h, and >7 to 24 h were analyzed for flavonoids and their metabolites by ultra-high-performance liquid chromatography-mass spectrometry (UPLC-MS/MS). Stool microbiome speciation was determined via Illumina sequencing. Linear mixed-effect models were used to assess differences in cumulative excretion across age and sex with time-by-group interaction taken as the principal analysis of effect.

**Results:**

There were no group (older females, older males, younger females, and younger males) differences in total 24 h urinary metabolite recovery, but there was a trend toward a higher rate of cumulative recovery in older males versus younger males at 24 h (*P*-group at 24h = 0.06). Of 76 metabolites, 20 had significantly different times of maximum urine excretion (Tmax) by age and 9 by sex, with a later mean Tmax observed for older participants (92% of instances). Associations with age were not mediated by body mass index (BMI) or microbiome speciation. Significant differences in maximum urine excretion (Cmax) by sex were observed for only 6 metabolites and differences by age for 5 metabolites.

**Conclusion:**

Total elimination recovery of (poly)phenols was relatively consistent across age and sex groups, whereas elimination kinetics (Tmax) differed substantially being much later in older indivudals, possibly resulting from differences in intestinal transit time or kidney clearance. Assuming (poly)phenol metabolites have varying biological activities, establishing dose-response relationships and defining metabolite profiles in population subgroups is required to inform the future development of dietary flavonoid/(poly)phenol recommendations.

This trial was registered at clinicaltrials.gov as NCT01922869

## Introduction

Flavonoids are a diverse group of dietary phytochemicals that are structurally subclassified into anthocyanins, flavonols, flavones, flavanones, flavan-3-ols, and isoflavones, and include their oligomeric (i.e., flavonoids) and polymeric (i.e., proanthocyanidins) forms [1,2]. Their bioactivity is, in part, responsible for the health benefits of fruits and vegetables and other plant-based foods, such as tea, fruit juice, wine, herbs, and spices [3,4]. In prospective cohort studies, a high flavonoid intake is associated with a 10%–40% reduction in all-cause mortality, dementia incidence, and cognitive decline, cardiovascular events, risk of specific cancers and, a lower body weight [[5], [6], [7], [8], [9]]. An accumulating number of randomized control trials (RCTs) are demonstrating, at a group level, clinically meaningful impacts of increased flavonoid intake on chronic disease biomarkers, providing insight into the underlying mechanisms of action at play [5,6,8,10]. The dose-response relationships between flavonoid consumption and phenotype are, however, highly variable, with large heterogeneity in the concentration of flavonoid metabolites in biological samples following intake of a given dose, as well as the biochemical and physiological responses observed [1,8,[10], [11], [12], [13]]. The molecular and physiological basis for this heterogeneity in dietary flavonoid metabolism and responsiveness observed across studies is poorly understood.

As reviewed, although a small number of existing studies have investigated potential modulators of (poly)phenol metabolism and elimination, most have had a limited number of participants, with heterogeneity in response being a secondary post hoc rather a priori aim, which often results in a poorly powered analysis [14,15].

Once ingested, the absorption, distribution, metabolism, and elimination of flavonoids is influenced by human intestinal, liver, and kidney functions, including phase 1 and phase 2 metabolizing enzymes, many of which are also important in the metabolism of xenobiotics and drugs [1,4]. Although not well characterized for flavonoid metabolism, there is emerging evidence to suggest that the functionality of these enzymes may be influenced by such physiological variables as sex and age [[16], [17], [18]]. Furthermore, a substantial fraction of flavonoid absorption, distribution, metabolism, and elimination is impacted by gut microbial metabolism and flavonoid intake has also been shown to influence microbiota speciation [19,20]. However, the role of gut microbiota functional and metabolic diversity in flavonoid metabolism and bioavailability is still poorly understood [1,21].

Here, using a prospective recruitment approach, we conducted the first systematic investigation of the impact of sex, age, and microbiota speciation on flavonoid elimination over 24 h following the consumption of a standardized cocoa/chocolate flavan-3-ol, citrus flavanone, and blackberry anthocyanin-rich test meal. Such establishment of metabolism and elimination kinetics in population subgroups is needed to inform and refine the development of dietary flavonoid recommendations [22].

## Methods

### Study participants

The acute chocolate, orange/citrus, blackberry intervention study (COB study) recruited prospectively on the basis of sex and age and aimed to include equal numbers of males and females and those aged between 18 and 30 y or 65 and 77 y who were generally healthy. Exclusion criteria were: BMI < 18.5 kg/m^2^ or > 30 kg/m^2^; smokers or nicotine users; hypertension with either a systolic blood pressure >140 mmHg or a diastolic blood pressure >90 mmHg; a medical condition or significant medical history likely to affect study measurements e.g., diagnosed type 2 diabetes, cardiovascular, renal, liver, thyroid or gastrointestinal diseases; vaccinations or antibiotics use in the previous 3 mo, (because these will impact on the gut microflora composition and metabolism and also phase 1 and 2 metabolism); blood biochemistry (including alanine aminotransferase >40 IU/L, alkaline phosphatase >130 IU/L, bilirubin, albumin, urea, and creatinine) outside the normal ranges; prescribed medication use that could interact with the enzymes involved in the metabolism of flavonoids (Supplementary Table S1); taking flavonoid containing supplements or other dietary supplements for 1 mo prior to the study; known allergies to the intervention foods; consume >21 alcohol unit/wk for males, or 14 units/wk for females; pregnant, lactating, or planning a pregnancy (or having fertility treatment); unable to provide informed consent to participate in the study.

Because excessively high intake of flavonoids has been shown to alter human metabolic pathways [23,24], individuals who are atypically high consumers of flavonoids as part of their regular diet were precluded from participating. At the time of the COB trial delivery, United Kingdom adults were reported to consume a mean intake of 10 servings of flavonoid-rich foods (including tea, coffee, chocolate, fruit, vegetables, and other plant-based products) per day (National Diet and Nutrition Survey, 2011) [25]. Individuals were excluded if they consumed >15 servings of flavonoid-rich foods per day. Flavonoid intake was assessed using the EPIC food frequency questionnaire [26].

### Study design

**FIGURE 1:**
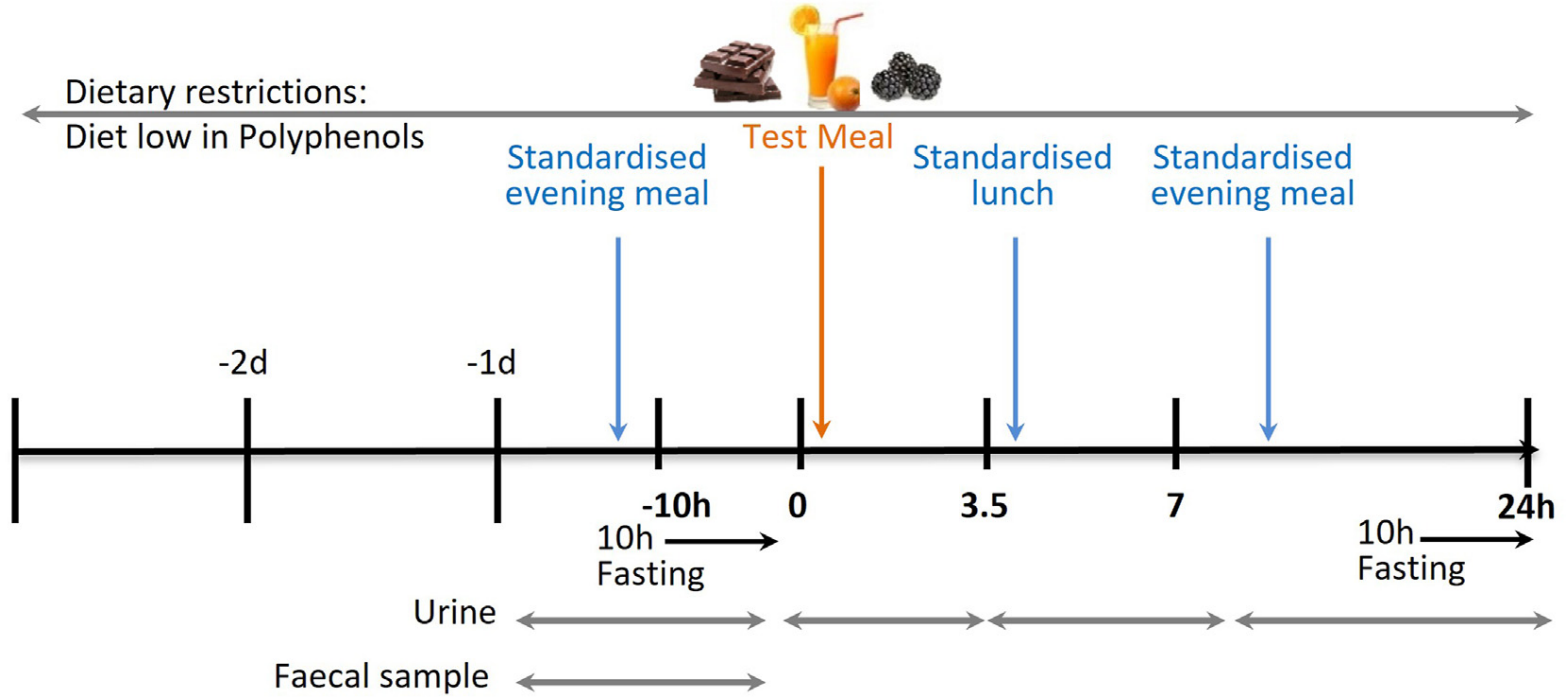
Chocolate, orange juice and blackberry (COB) intervention to examine flavonoid metabolism.

During the dietary run-in period and acute test-meal protocol, participants were asked to adhere to a restricted diet to minimize the intake of (poly)phenols (including flavonoids and phenolics) by avoiding the consumption of fruits, vegetables, chocolate, spices, high-fiber products, tea, coffees, fruit juices, and alcoholic beverages (Supplementary Table S2) for 48 h before the study day and for the 24 h after the consumption of the flavonoid-rich test meal (Figure 1). In addition to the medications listed in Supplementary Table S1, participants were asked to avoid acetaminophen (paracetamol) or other nonsteroidal anti-inflammatory drugs for the 10 h (fasting period) prior to the acute study day and for the duration of the sample collection unless it was a medical requirement.

Participants were provided with a standardized low-flavonoid ready meal, consisting of either a vegetable-free fish pie or macaroni cheese, along with a bread roll and a portion of fruit-free sponge cake to consume the evening prior to attending the clinic visit in order to control for any interindividual variability in background dietary (poly)phenols from their previous meals, and then undergo an overnight fast (only water allowed during this fasting period). The participants attended the clinical trial unit after an overnight fast for the acute test-meal session containing mixed flavonoids. The standardized mixed flavonoid-rich test meal consisted of a bar of dark chocolate (Barry Callebaut, 50 g), a freeze-dried orange extract (Monteloeder, 362 mg), and blackberry (Nutra Ingredients Ltd, 37.5 g) powder in water, known as the COB mixture, with the composition of the chocolate provided by the manufacturer (Barry Callebut, Belgium) and the flavonoid composition of the blackberry powder and citrus extracts established using an internal UHPLC-MS/MS based analysis [10,27,28]. This provided 640-mg flavan-3-ols (∼30% monomers, ∼180 mg (-)-epicatechin), 340-mg flavanones (89.3% hesperitin, 3.4% narirutin), and 390-mg anthocyanins (99+% cyanidin-3-*O*-glucoside). Urine samples were collected at baseline (−24 to 0 h, before the test meal), 0–3.5 h, 3.5–7 h, and 7 –24 h. Participants were free to leave the clinical trial unit at 7 h and return at the 24 h points for the relevant urine sampling. During the day of the intervention, 2 standardized low-flavonoid meals (lunch and dinner) were provided along with low-flavonoid snacks (Figure 1).

The study was approved by the National Health Service Health Research Authority (IRAS Project ID 1251207), followed the principles of the Declaration of Helsinki, and was conducted at the Clinical Research Facility, University of East Anglia. Informed consent was obtained from all participants before study commencement. Recruitment occurred between October 2013 and March 2015, with follow-up completed by April 2015. Clinical Trials Registration # NCT01922869.

### Dietary assessment

Participants completed a 131-item validated [26] food frequency questionnire (FFQ), which captured dietary habits over the previous 12 mo, from which nutrient intakes were determined using McCance and Widdowson Food Tables [29,30] and flavonoid intakes were calculated using the updated USDA databases for the flavonoid and proanthocyanin content of food, as previously described [31]. If no values were available in the USDA database (USDA Database for the Flavonoid Content of Selected Foods Release 3.1) for foods in the FFQ, available data from the Phenol-Explorer database (www.phenol-explorer.eu) [32,33] were included. Flavonoid intakes were derived for the 6 main flavonoid subclasses habitually consumed: flavanones (eriodictyol, hesperetin, and naringenin); anthocyanins (cyanidin, delphinidin, malvidin, pelargonidin, petunidin, and peonidin); flavan-3-ols (catechins and epicatechins); flavonols (quercetin, kaempferol, myricetin, and isorhamnetin); flavones (luteolin and apigenin); and polymers (including proanthocyanidins, theaflavins, and thearubigins). Total flavonoid intakes were estimated by summing the 6 component subclasses.

### Analytical methods

Total urine voids were collected into light-protected collection bottles containing 1 g of ascorbic acid. Aliquots were stored at −80° C until analysis. Urinary metabolites were purified from 50 μL human urine (1 mL aliquots acidified with 40 μL formic acid) samples by 96-well plate solid phase extraction (SPE; Strata-X Polymeric Reversed Phase, microelution 2 mg/well). Taxifolin was spiked into the urine and used as an SPE recovery reference standard, and scopoletin post-SPE as an internal chromatography standard. The solid phase extraction treated samples were chromatographically separated on an Exion ultrahigh performance UHPLC coupled to a SCIEX QTRAP 3200+ triple quadrupole mass spectrometer (MS/MS; SCIEX, Framingham) with electrospray ionization source, as previously reported [10,27,28]. The samples were injected into Kinetex (Phenomenex) 2.6 μm pentafluorophenyl phase (PFP) 100 Å, LC Column 100 × 4.6 mm (Part Number: 00D-4477-E0) with SecurityGuard ULTRA cartridges for PFP, with oven temperature maintained at 37°C. Mobile phase A and B consisted of 0.1% v.v. formic acid in water and 0.1% v.v. formic acid in acetonitrile respectively, with a binary gradient ranging from 1% B to 90% B over 28 min and flow rate at 1.5 mL/min. MS/MS scanning was accomplished using a targeted advanced scheduled multiple reaction monitoring assay using polarity switching between positive and negative ionization mode in Analyst (v.1.6.3, SCIEX) and with quantitation conducted using MultiQuant (v.3.0.2, SCIEX) software platforms. If 2 metabolite isomers could not be resolved, they were quantified relative to a single species. Finally, metabolites previously reported in nutrition intervention studies feeding (poly)phenol-rich foods [27,28,[34], [35], [36], [37], [38], [39], [40], [41], [42], [43], [44], [45]] were confirmed on the basis of established retention times (using authentic and synthesized standards; Supplementary Table S3) and >3 precursor-to-product transition ions. In total, 82 (poly)phenols were quantified via UPLC-MS/MS; 6 metabolites were below the limit of quantitation in all samples, with statistical analysis completed for 76 analytes. All the metabolites were quantified relative to their reference standard, with the exception of cyanidin-diglucuronide, which was quantified using the cyanidin-3-O-glucoside reference standard. Matrix-matched standard curves were prepared for quantification ranging from 0–10 μM. Reference standards scopoletin, taxifolin, and phloridzin were used as quality controls and for internal standard adjustment in the urine samples, with system blanks monitored for carryover effects and preextracted urine as a reference blank.

Stool microbiome speciation. Stool specimens were collected on the day prior to the study day using EasySampler kits (GP Medical Devices, Nupark), transferred into sterile containers (Sarstedt), transported in cooled bags provided by the research team, and immediately transferred to a −20°C freezer on arrival. Prior to DNA extraction, 200–300 mg feces was transferred to a 2 mL safe lock tube (Eppendorf) containing a 5 mm steel ball bearing and was subjected to mechanical disruption in 1.2 mL sterile phosphate-buffered saline using a tissue lyser (Qiagen Tissue Lyser II, 30 Hz for 4 min at 4°C). Samples were centrifuged to remove insoluble material (1 min, 10,000 × *g* at 4°C). DNA was extracted from 800 μL of the supernatant using a QIAsymphony SP automated platform (Qiagen) using the QIAsymphony DSP virus/bacteria midi kit and the Complex 400 V6 DSP program. DNA was eluted in 110 μL buffer AVE containing carrier RNA as provided in the kit. The DNA was quantitated to confirm a concentration between 5–20 ng/μL and stored at −20°C until analysis. The region of the 16S gene was sequenced at APHA, Surrey (UK), using paired-end Illumina MiSeq short-read sequencing. The Qiime [46] 1.9.0 Illumina workflow was used for joining the reads, demultiplex, and filter samples and picking operational taxonomic units. Read counts varied across the samples, from 70 in a control sample to 112,944 in sample COB354. Sequencing control samples were 2 separate phosphate-buffered saline samples and 1 sample with no template. Two samples were excluded based on having a sequencing depth below the threshold. In addition, Qiime excluded 2 more samples according to the quality control filter. This filter is described in Bokulich et al. [47]. Qiime alpha diversity analyses (secondary analysis) involved testing a range of multiple rarefactions run in parallel. The resulting graph from these technical analyses did not indicate large-scale differences from using data at different sequencing depth levels (data not shown).

### Statistical analysis

Given the absence of established effect size estimates at the time the intervention was designed, we justified our sample size based on prior empirical data from Czank et al. [11], where significant changes in polyphenol metabolites were observed in 8 participants. Based on the observed confidence intervals in that study, we estimated that group sizes of 30–40 participants would be sufficient for age and sex subgroup analyses. In the analysis of metabolites, values above the signal-to-noise but below the limit of detection (LOD) were set as zero in the statistical analysis. If metabolites had the majority of their values at 0, they were excluded from the analysis. Calibration curves were established between 1nM and 10 uM. Values below the lower limit of quantitation but above the LOD were reported as a “near 0” value (0.0001) in the statistical analysis. Linear mixed-effect models were used to assess differences in cumulative excretion by age (younger compared with older), sex (males compared with females), and combined age-by-sex (younger females, younger males, older females, and older males) groups. Models included cumulative excretion as the dependent variable, “participant” as a random effect, time (0 min, 210 min, 420 min, and 1440 min), and groups as predictors, with the time x group interaction taken as the principal analysis of effect. The linear combinations of coefficients between groups were explored at the 24 h timepoint. Total flavonoid intake, as calculated from the FFQ, was included as a covariate on a continuous scale (where indicated) to explore if habitual diet markedly changed the results. Data were not transformed as generalized linear models have shown to be tolerant of distribution assumptions and can provide valid inference regardless of the distribution of the data [48]. Total recovery of the metabolite mass in a urine sample is calculated by converting its molar concentration (moles per volume) to mass (e.g., ng) using the molecular weight of the metabolite, then multiplying by the total urine volume excreted: (Metabolite value [nmol/L] × molecular weight [ng/nmol] /1,000,000)×(urine volume [L]))/time [h]. Where indicated in data tables, low concentrations are presented as 10^−^^2^ (for example, 0.01×10^−^^2^ = 0.0001 ng). The UPLC-MS/MS standard mix contained 82 (poly)phenols (i.e., comprising both precursor polyphenols and phenolic metabolites; Table S3), and 76 were above the lower limit of quantitation in the COB urine samples. Six metabolites were found below the LOD in the majority of samples and were, therefore, not included in the final statistical analysis. Elimination kinetic parameters were calculated for each metabolite, maximum urine concentration (Cmax), time of maximum concentration (Tmax) (0 h, 3.5 h, 7 h, or 24 h), and total amount excreted in urine over 24 h (Aeu 0-24). Differences in Cmax and Tmax were compared between groups using a linear regression model with the elimination parameter as the dependent variable, group as the predictor, and habitual total flavonoid intake on a continuous scale as a covariate (where indicated). If metabolite data were missing at individual time points, cumulative excretion was calculated based on the missing data point. Cmax and Tmax were calculated if data were available for at least one timepoint. *P* values < 0.05 were considered statistically significant. The Benjamini-Hochberg method for false discovery rate (using a false discovery rate Q-value < 0.20) was also used to explore the possible impact of multiple testing on observed age and sex effects. Statistical analyses were performed with Stata statistical software version 16 (StataCorp).

Linear discriminant analysis was used to identify which combination of metabolites separated the participants by group and how effective these metabolites were as predictive discriminators of age. Metabolites included in the model were those with a significant time-by-age group interaction in the primary analysis.

Differences in relative abundance of taxa at the Family level and microbial alpha diversity (Shannon Index) were compared across age (younger compared with older), sex (males compared with females), and combined age-by-sex (younger females, younger males, older females, and older males) groups using linear regression with group as the predictor. We excluded taxa where relative abundance was < 0.01% in ≥10% of samples, leaving 38 taxa in the final analysis.

Hierarchical regression analysis (using metabolite concentration as the dependent variable) was used to examine if models including sex and microbial composition improved prediction of metabolite concentrations over models including age. For microbial composition, we combined the taxa significantly associated with age using principal component analysis, considering the first component defined. The metabolites included in the model were those with a significant time-by-group interaction in the primary analysis.

## Results

**FIGURE 2:**
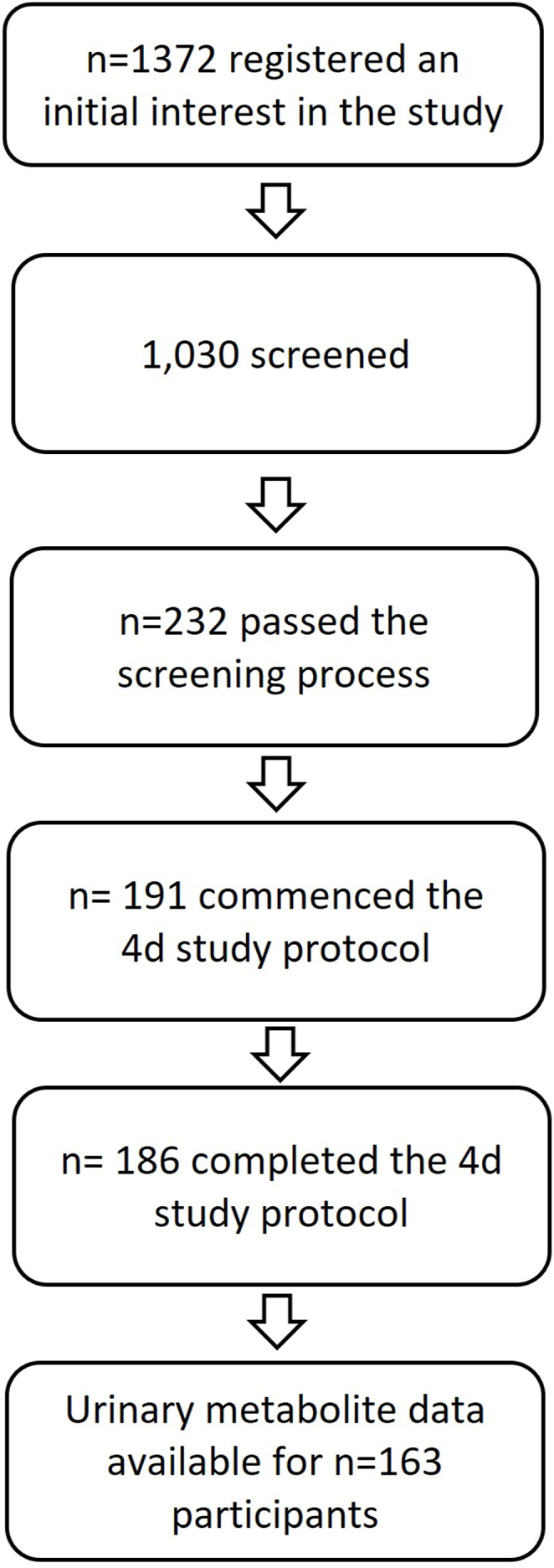
Participant flow diagram.

A total of 186 participants completed the COB protocol (Figure 2). Metabolite concentrations in urine were quantified in 163 participants (44 older females, 46 older males, 42 younger females, and 31 younger males) for 24 h, and data on habitual diet was available for 161 participants (Table 1). Older males had the highest caloric intake (age and sex effect; *P* < 0.04 and 0.03, respectively), and BMI was higher in older participants and highest in older males (*P* < 0.01). Older participants consumed considerably more flavonoids (*P* < 0.01), with nearly 3 times higher habitual intakes than younger individuals, which was reflected in higher total fruit, vegetable, and tea intakes. No notable differences in flavonoid intakes were observed by sex, whereas females reported lower total energy and carbohydrate intakes and a higher fruit and vegetable intake.

**TABLE 1:** Participant characteristics mean (95% CI) and habitual dietary intake stratified by age and sex group in 163 participants from the COB study

	Older females	Older males	Younger females	Younger males
	(*n* = 44)	(*n* = 46)	(*n* = 42)	(*n* = 31)
Age (y)	66.9 (65.5, 68.2)	67.8 (66.5, 69.1)	22.6 (21.2, 24.0)	22.4 (20.8, 24.0)
BMI (kg/m^2^)	24.2 (23.4, 25.0)	25.4 (24.6, 26.3)	23.4 (22.6, 24.3)	23.0 (22.0, 24.0)
Energy (kcal)	2063 (1861, 2265)	2229 (2036, 2422)	1811 (1609, 2013)	2079 (1844, 2315)
Protein (g)	89.7 (81.7, 97.7)	89.9 (82.3, 97.6)	78.2 (70.3, 86.2)	88.9 (79.6, 98.2)
Fat (g)	74.5 (65.9, 83.0)	78.5 (70.3, 86.7)	62.6 (54.1, 71.2)	75.5 (65.5, 85.5)
Carbohydrate (g)	258 (229, 286)	287 (259, 314)	241 (212, 269)	269 (236, 303)
Fiber (g)	25.5 (22.8, 28.3)	25.4 (22.8, 28.0)	22.9 (20.1, 25.6)	22.7 (19.5, 25.9)
Total flavonoids (mg/d)	1298 (1150, 1446)	1237 (1096, 1378)	509 (361, 657)	478 (306, 650)
Flavanones (mg/d)	41.1 (31.8, 50.4)	30.8 (22.0, 39.7)	23.4 (14.1, 32.7)	22.1 (11.3, 33.0)
Anthocyanins (mg/d)	34.7 (29.1, 40.3)	29.5 (24.2, 34.9)	19.2 (13.6, 24.8)	11.5 (5.0, 18.0)
Flavan-3-ols (mg/d)	254 (220, 289)	255 (222, 288)	102 (67.6, 136)	84.8 (44.8, 125)
Flavonols (mg/d)	57.1 (51.1, 63.0)	53.2 (47.5, 58.9)	29.7 (23.7, 35.6)	24.6 (17.7, 31.6)
Flavones (mg/d)	1.9 (1.6, 2.2)	1.7 (1.4, 1.9)	1.6 (1.3, 1.8)	1.2 (0.89, 1.5)
Polymers (mg/d)	909 (801, 1017)	866 (763, 969)	333 (225, 441)	334 (208, 459)
Fruit and vegetables (g/d)	794 (696, 891)	653 (559, 746)	583 (486, 681)	481 (367, 594)
Tea (g/d)	763 (650, 877)	759 (650, 869)	204 (88.9, 319)	217 (76.1, 358)

Values are mean (95% CI). *n* = 163 (data missing for *n* = 2 participants for dietary data).

### Cumulative Excretion: sum of all (poly)phenols and metabolites

There was a trend for age-by-sex differences in cumulative (all urine “time bins”) 24 h urinary metabolite recovery (*P* = 0.06; treatment effect, all metabolites and parent (poly)phenols; Supplementary Table 4); however, no significant time-by-group interactions were observed (*P* = 0.30); with mean total urinary recovery of 14.59 ± 2.22 ng at baseline, 56.02 ± 7.72 ng at 3.5 h, 97.80 ± 20.73 ng at 7 h; and 122.32 ± 25.34 ng at 24 h (mean ± SD; data not shown). There were also no significant group differences (mean, 95% confidence interval [CI]) for maximum total excretion (Cmax, *P* = 0.20) [(older females 505 ng (333, 678), older males 670 ng (502, 839), younger females 452 ng (276, 629), younger males 384 ng (179, 590)]; however, there was significant group differences in Tmax (*P* = 0.04) (older females 15.8 h [12.4, 19.2], older males 15.2 h [11.9, 18.5)], younger females 10.6 h [7.1, 14.1], and younger males 12.0 h [7.9, 16.0]; [mean, 95% CI]).

### Individual (poly)phenols and metabolite excretion

Differences in cumulative 24 h excretion were observed for 12 individual metabolites by age (time-by-treatment interaction, *P* < 0.05; 10 Q < 0.2) and 5 metabolites by sex (1 Q < 0.2) (Table 2; Complete dataset found in Supplementary Table S4 ). Here, the metabolites primarily comprised of small molecule microbial metabolites of (poly)phenols, where younger participants, mainly males, generally displayed higher cumulative excretion. Adjustment for habitual total flavonoid intake did not materially change the age differences observed in urinary excretion over time or cumulative 24-h urinary recovery (Supplementary Table S5).

**TABLE 2:** Cumulative excretion (ng) by age and sex group at 24 h

Metabolite (ng)	Older females		Older males		Younger females		Younger males		*P* group	*P* age	*P* sex
	*n*	Mean (95% CI)	*n*	Mean (95% CI)	*n*	Mean (95% CI)	*n*	Mean (95% CI)			
3-(dihydroxyphenyl)propionic acid^2^	42	0.043 (0.034, 0.053)	45	0.035 (0.025, 0.044)	42	0.056 (0.046, 0.065)	28	0.046 (0.035, 0.058)	0.02^1^	0.01^1^	0.05
4-methylhippuric acid (x 10^2^)	42	0.297 (0.173, 0.421)	45	0.629 (0.509, 0.749)	42	0.296 (0.171, 0.421)	29	0.412 (0.263, 0.562)	<0.01^1^	0.07	<0.01^1^
3-methoxycinnamic acid	42	0.033 (0.009, 0.056)	45	0.079 (0.056, 0.101)	42	0.049 (0.025, 0.072)	29	0.024 (-0.004, 0.052)	<0.01^1^	0.15	0.18
6-methoxysalicyclic acid (x 10^2^)	42	0.091 (0.031, 0.150)	45	0.225 (0.166, 0.283)	41	0.147 (0.086, 0.208)	29	0.057 (-0.016, 0.129)	<0.01^1^	0.12	0.22
4-hydroxy-3-methoxyacetophenone	42	0.020 (0.013, 0.028)	45	0.029 (0.022, 0.037)	42	0.036 (0.028, 0.044)	29	0.037 (0.028, 0.046)	0.01^1^	<0.01^1^	0.28
benzoylglutamic acid	42	0.012 (0.008, 0.016)	45	0.021 (0.017, 0.025)	42	0.013 (0.009, 0.018)	29	0.013 (0.007, 0.018)	0.01^1^	0.11	0.03
5-(hydroxyphenyl)-gamma-valerolactone-sulfate	42	0.313 (0.192, 0.434)	45	0.585 (0.468, 0.703)	42	0.506 (0.385, 0.627)	29	0.186 (0.040, 0.331)	<0.01^1^	0.23	0.77
5-(hydroxyphenyl)-gamma-valerolactone (x 10^2^)	42	2.4 (1.4, 3.4)	44	2.2 (1.3, 3.2)	42	4.1 (3.1, 5.0)	28	1.4 (0.207, 2.6)	<0.01^1^	0.19	0.01^1^
naringenin-7-*O*-glucuronide (x 10^2^)	42	3.4 (2.5, 4.4)	45	4.2 (3.3, 5.1)	42	4.9 (4.0, 5.9)	29	2.4 (1.2, 3.5)	<0.01^1^	0.88	0.17
2,3-dihydroxybenzoic acid	42	0.059 (0.038, 0.080)	45	0.101 (0.081, 0.121)	42	0.093 (0.072, 0.114)	29	0.067 (0.042, 0.092)	0.01^1^	0.86	0.29
3-methylhippuric acid	42	0.006 (0.004, 0.009)	45	0.014 (0.012, 0.016)	42	0.006 (0.004, 0.009)	29	0.005 (0.002, 0.008)	<0.01^1^	<0.01^1^	<0.01^1^
3-methoxybenzoic acid-4-sulfate	41	0.616 (0.441, 0.791)	42	0.870 (0.697, 1.0)	41	0.525 (0.348, 0.701)	29	0.900 (0.691, 1.1)	<0.01^1^	0.50	<0.01^1^
benzoic acid-4-*O*-glucuronide	42	0.034 (0.029, 0.040)	45	0.028 (0.023, 0.033)	42	0.028 (0.022, 0.033)	29	0.017 (0.011, 0.024)	<0.01^1^	0.01^1^	0.02^1^
2-hydroxycinnamic acid (x 10^2^)	42	0.021 (0.007, 0.035)	46	0.018 (0.005, 0.032)	41	0.022 (0.008, 0.036)	30	0.065 (0.049, 0.082)	<0.01^1^	<0.01^1^	0.04
3-(4-hydroxy-3-methoxyphenyl)propionic acid	42	0.051 (0.026, 0.075)	46	0.081 (0.058, 0.105)	41	0.106 (0.081, 0.130)	30	0.106 (0.077, 0.135)	<0.01^1^	<0.01^1^	0.31
hesperetin-3'-*O*-glucuronide	42	0.517 (0.346, 0.689)	46	0.772 (0.607, 0.937)	41	0.567 (0.393, 0.741)	30	0.356 (0.152, 0.559)	0.02^1^	0.06	0.48
3-methoxy-4-hydroxyphenylacetic acid	42	0.922 (0.530, 1.3)	46	1.7 (1.3, 2.1)	41	1.0 (0.605, 1.4)	30	0.842 (0.378, 1.3)	<0.01^1^	0.06	0.06
3-methoxybenzoic acid-4-*O*-glucuronide	42	0.394 (0.236, 0.552)	46	0.625 (0.473, 0.777)	41	0.462 (0.302, 0.623)	30	0.285 (0.098, 0.471)	0.04^1^	0.13	0.46
hydroxy-methoxybenzoic acid^2^	42	0.065 (0.045, 0.085)	46	0.075 (0.056, 0.095)	41	0.090 (0.069, 0.110)	30	0.133 (0.109, 0.157)	<0.01^1^	<0.01^1^	0.06
hydroxybenzoic acid^2^	42	0.010 (0.007, 0.013)	46	0.010 (0.008, 0.013)	40	0.013 (0.010, 0.016)	30	0.005 (0.002, 0.009)	<0.01^1^	0.78	0.05
3-hydroxyhippuric acid	42	1.8 (1.3, 2.2)	46	2.5 (2.0, 2.9)	41	1.5 (0.986, 1.9)	30	1.6 (1.1, 2.2)	0.02^1^	0.02^1^	0.04
4-methoxybenzaldehyde	41	0.007 (0.005, 0.009)	46	0.005 (0.003, 0.007)	41	0.008 (0.007, 0.010)	29	0.011 (0.008, 0.013)	<0.01^1^	<0.01^1^	0.59
3-hydroxy-4-methoxybenzoic acid	42	0.096 (0.074, 0.119)	46	0.082 (0.061, 0.104)	41	0.140 (0.117, 0.163)	29	0.166 (0.139, 0.193)	<0.01^1^	<0.01^1^	0.82
hydroxybenzoic acid-sulfate^2^	39	0.318 (0.188, 0.447)	44	0.544 (0.421, 0.667)	39	0.303 (0.173, 0.433)	26	0.404 (0.245, 0.563)	0.03^1^	0.18	<0.01^1^
hippuric acid	34	58.1 (44.9, 71.3)	37	77.8 (65.1, 90.5)	34	58.7 (45.4, 72.0)	22	45.6 (29.2, 61.9)	0.02^1^	0.04	0.31
3-(phenyl)propionic acid	34	0.019 (-0.020, 0.058)	38	0.009 (-0.028, 0.046)	34	0.047 (0.008, 0.086)	22	0.092 (0.044, 0.140)	0.04^1^	0.01^1^	0.75

Values are mean (95% CI). Metabolites shown are those with a significant group, age, or sex effect. *P* values are for group comparisons at 24 h and calculated from linear mixed-effect models.

1 False discovery rate adjusted *P* values < 0.2. Full dataset providing *P* values for all analytes is found in Supplement Table S4.

2 Metabolite isomers that could not be resolved effectively by HPLC were quantified according to one of the structural isomers. All urine metabolites values are presented as: {[Metabolite value (nmol/L) × molecular weight (ng/nmol) /(1,000,000) × (urine volume L)]}/time (h). Where indicated in data tables, low concentrations are presented as 10ˆ2 (for example, 0.01 × 10^-^2 = 0.0001 ng).

### Elimination kinetics

Differences in Cmax were observed for 7% of measured metabolites by age and 8% by sex, whereas differences in Tmax were observed for 28% by age and 11% of metabolites by sex (Supplementary Table S6; 36 *P* < 0.05; 14 Q < 0.2), with the majority of differences seen as age effects on Tmax.

Group differences in Cmax were observed for 4 metabolites (*P* < 0.05; 2 Q < 0.2), with the highest Cmax observed most frequently in older males (Table 3; complete dataset found in Supplementary Table S6). Age differences were observed for 5 phenolic metabolites (0 Q < 0.2) and sex differences for 6 metabolites (0 Q < 0.2). Metabolites recorded in highest concentrations (Cmax; mean [95% CI]) having age or sex differences were 3-hydroxyhippuric acid 22.0 μM (14.7, 29.3), benzoic acid-4-sulfate 17.3 μM (12.7, 21.8), 3-methoxybenzoic acid-4-sulfate 4.4 μM (3.1, 5.8), 2,5-dihydroxybenzoic acid 0.85 μM (0.53, 1.2), 3-hydroxy-4-methoxybenzoic acid 0.60 μM (0.46, 0.74), hydroxy-methoxybenzoic acid 0.42 μM (0.32, 0.52), and 4-methylhippuric acid 0.03 μM (0.01, 0.04). Adjusting Cmax for total flavonoid intake by age group had a limited impact on the statistical output (Supplementary Table S7).

**TABLE 3:** Maximum urinary elimination (Cmax) by age and sex group

Metabolite (ng)	Older females		Older males		Younger females		Younger males		*P* group	*P* age	*P* sex
	*n*	Mean (95% CI)	*n*	Mean (95% CI)	*n*	Mean (95% CI)	*n*	Mean (95% CI)			
2,5-dihydroxybenzoic acid	44	0.52 (0.26, 0.79)	46	0.83 (0.58, 1.1)	42	0.54 (0.27, 0.81)	31	0.85 (0.53, 1.2)	0.34	0.95	0.03
4-methylhippuric acid	44	0.02 (0.01, 0.03)	46	0.05 (0.04, 0.06)	42	0.02 (0.003, 0.03)	31	0.03 (0.01, 0.04)	0.53	0.03	<0.01^1^
3-methylhippuric acid	44	0.05 (0.02, 0.07)	46	0.11 (0.08, 0.13)	42	0.04 (0.01, 0.07)	31	0.04 (0.01, 0.07)	0.29	0.02	0.02
3-methoxybenzoic acid-4-sulfate	44	1.8 (0.68, 2.9)	46	2.7 (1.6, 3.8)	42	1.4 (0.29, 2.6)	31	4.4 (3.1, 5.8)	0.04	0.45	<0.01^1^
benzoic acid-4-*O*-glucuronide	44	0.16 (0.13, 0.20)	46	0.14 (0.10, 0.17)	42	0.13 (0.09, 0.17)	31	0.09 (0.05, 0.14)	0.02	0.07	0.13
benzoic acid-4-sulfate	44	9.7 (5.0, 14.3)	46	17.3 (12.7, 21.8)	41	8.1 (3.3, 12.9)	31	10.8 (5.3, 16.4)	0.62	0.09	0.02
hydroxy-methoxybenzoic acid^2^	44	0.17 (0.09, 0.26)	46	0.25 (0.17, 0.33)	41	0.26 (0.18, 0.35)	31	0.42 (0.32, 0.52)	<0.01^1^	0.01	0.02
3-hydroxyhippuric acid	44	17.5 (10.0, 25.0)	46	22.0 (14.7, 29.3)	41	10.5 (2.7, 18.2)	31	12.6 (3.6, 21.5)	0.14	0.03	0.30
3-hydroxy-4-methoxybenzoic acid	44	0.28 (0.16, 0.39)	46	0.29 (0.18, 0.41)	42	0.37 (0.25, 0.48)	31	0.60 (0.46, 0.74)	<0.01^1^	<0.01	0.12

Values are mean (95% CI). Metabolites shown are those with a significant group, age or sex effect; *P* values calculated from linear regression.

1 false discovery rate adjusted *P* values < 0.2. Full dataset providing *P* values for all analytes is found in Supplement Table S6.

2 Metabolite isomers which could not be resolved effectively by HPLC were quantified according to one of the structural isomers. All urine metabolites values are presented as: {[Metabolite value (nmol/L) × molecular weight (ng/nmol)/1000000] × [urine volume (L]}/time (h).

The time to maximum urinary concentration (Tmax) varied considerably across metabolites, displaying group, age, or sex differences, from 1h and 24 h, with a mean Tmax of 9.1 ± 4.1 h [mean (95% CI); Table 4]. Group differences were observed for 15 metabolites (4 Q > 0.2). Twenty-two of the metabolites displayed significant Tmax differences by age (14 Q < 0.2) and 8 by sex (0 Q < 0.2), with 92% of these metabolites reflecting significantly later Tmax in older participants. Only 2 analytes, 4-hydroxybenzyl alcohol and 3-methylhippuric acid, had greater Tmax in younger participants (all analyte data provided in Supplementary Table S6). Adjusting Tmax for total flavonoid intake by age group had limited impact on the statistical output (Supplementary Table S7).

**TABLE 4:** Time (h) of maximal urinary elimination (Tmax) by age and sex group

Metabolite	Older females		Older males		Younger females		Younger males		*P* group	*P* age	*P* sex
	*n*	Mean (95% CI)	*n*	Mean (95% CI)	*n*	Mean (95% CI)	*n*	Mean (95% CI)			
3,4-dihydroxyphenylacetic acid	44	14.7 (11.4, 17.9)	46	15.7 (12.5, 18.8)	42	16.3 (12.9, 19.6)	31	20.9 (17.1, 24.8)	0.02	0.08	0.18
3-methoxycinnamic acid	44	8.1 (4.8, 11.4)	46	13.3 (10.0, 16.5)	42	6.9 (3.5, 10.3)	31	9.5 (5.6, 13.5)	0.76	0.12	0.02
4-hydroxybenzaldehyde	44	11.3 (8.0, 14.6)	46	13.5 (10.3, 16.7)	42	5.5 (2.1, 8.9)	31	9.3 (5.4, 13.2)	0.06	<0.01^1^	0.06
4-hydroxybenzyl alcohol	44	12.0 (8.6, 15.4)	46	11.2 (7.9, 14.5)	42	13.6 (10.1, 17.1)	31	17.5 (13.4, 21.5)	0.04	0.05^1^	0.60
3-caffeoylquinic acid	44	14.0 (10.7, 17.2)	46	16.8 (13.6, 20.0)	42	13.1 (9.7, 16.4)	31	17.7 (13.8, 21.6)	0.44	0.82	0.03
hesperetin	44	11.8 (9.0, 14.5)	46	9.7 (7.0, 12.4)	42	11.1 (8.2, 13.9)	31	5.7 (2.4, 9.0)	0.03	0.20	0.02
hesperetin-7'-sulfate	44	5.9 (3.4, 8.4)	46	5.5 (3.1, 8.0)	42	2.2 (-0.3, 4.8)	31	3.1 (0.15, 6.1)	0.04	0.02^1^	0.74
3,4-dihydroxybenzoic acid methyl ester	44	5.5 (2.8, 8.2)	46	8.3 (5.7, 10.9)	42	2.6 (-0.1, 5.4)	31	6.0 (2.8, 9.2)	0.45	0.05^1^	0.02
naringenin-7-*O*-glucuronide	44	11.1 (8.5, 13.8)	46	13.3 (10.7, 15.9)	42	8.5 (5.7, 11.2)	31	10.2 (7.1, 13.4)	0.22	0.03^1^	0.12
naringenin-7-*O*-glucuronide	44	11.1 (8.5, 13.8)	46	13.3 (10.7, 15.9)	42	8.5 (5.7, 11.2)	31	10.2 (7.1, 13.4)	0.22	0.03^1^	0.12
3,4-dihydroxybenzoic acid	44	12.4 (9.4, 15.3)	46	12.4 (9.5, 15.3)	42	9.4 (6.3, 12.4)	31	7.7 (4.2, 11.2)	0.02	0.02^1^	0.81
4-hydroxycinnamic acid	44	6.4 (3.9, 8.9)	46	9.7 (7.3, 12.2)	42	5.4 (2.9, 8.0)	31	7.7 (4.7, 10.7)	0.90	0.20	0.02
trihydroxybenzaldehyde^2^	44	6.9 (5.1, 8.8)	46	7.9 (6.1, 9.7)	42	4.4 (2.5, 6.3)	31	4.8 (2.6, 7.1)	0.03	<0.01^1^	0.34
3-methylhippuric acid	44	4.4 (1.3, 7.4)	46	4.2 (1.2, 7.2)	42	7.1 (4.0, 10.2)	31	9.3 (5.7, 12.9)	0.02	0.02^1^	0.74
2-hydroxy-4-methoxybenzoic acid	44	5.9 (3.3, 8.4)	46	5.5 (3.0, 8.0)	42	2.3 (-0.3, 4.9)	31	2.3 (-0.7, 5.4)	0.02	0.01^1^	0.93
3-(3-hydroxyphenyl)propionic acid	44	21.3 (18.5, 24.0)	46	19.6 (16.9, 22.3)	42	20.6 (17.8, 23.4)	31	14.0 (10.8, 17.3)	0.01^1^	0.08	0.02
(-)-epicatechin	44	9.6 (6.8, 12.3)	46	9.7 (6.9, 12.4)	42	6.9 (4.1, 9.7)	31	5.8 (2.5, 9.1)	0.04	0.03^1^	0.92
4-hydroxybenzoic acid-3-*O*-glucuronide	44	5.9 (3.8, 8.0)	46	8.7 (6.6, 10.8)	42	4.2 (2.1, 6.4)	31	7.6 (5.0, 10.1)	0.94	0.14	0.01
hydroxy-methoxybenzoic acid^2^	44	8.6 (5.9, 11.2)	46	12.1 (9.5, 14.7)	42	6.3 (3.6, 9.1)	31	5.9 (2.7, 9.1)	0.04	<0.01^1^	0.14
hydroxybenzoic acid^2^	44	15.4 (12.0, 18.7)	46	13.8 (10.6, 17.1)	42	11.3 (7.9, 14.7)	31	10.3 (6.3, 14.3)	0.03	0.04^1^	0.58
3-hydroxybenzoic acid-4-*O*-glucuronide	44	9.6 (7.1, 12.0)	46	11.5 (9.0, 13.9)	42	6.3 (3.8, 8.8)	31	4.9 (2.0, 7.9)	<0.01^1^	<0.01^1^	0.52
cyanidin-3-*O*-glucoside	44	6.6 (4.5, 8.7)	46	6.7 (4.6, 8.7)	42	2.8 (0.60, 4.9)	31	6.4 (3.9, 8.9)	0.25	0.04^1^	0.10
ellagic acid	44	15.6 (12.7, 18.4)	46	15.1 (12.3, 17.9)	42	10.0 (7.1, 12.9)	31	13.4 (10.1, 16.8)	0.06	0.01^1^	0.29
3-hydroxy-4-methoxybenzoic acid	44	9.8 (6.8, 12.8)	46	11.4 (8.5, 14.3)	42	7.5 (4.4, 10.5)	31	7.1 (3.6, 10.7)	0.11	0.04^1^	0.51
catechin^2^	44	10.1 (7.0, 13.3)	46	12.3 (9.2, 15.4)	42	7.2 (4.0, 10.5)	31	8.6 (4.9, 12.4)	0.20	0.04^1^	0.21
hydroxybenzoic acid-sulfate^2^	44	9.6 (7.0, 12.1)	46	14.0 (11.5, 16.5)	42	5.6 (3.0, 8.3)	31	6.3 (3.2, 9.3)	0.01^1^	<0.01^1^	0.02
4-hydroxy-3-methoxybenzoic acid methyl ester	44	13.0 (9.8, 16.3)	46	9.3 (6.1, 12.4)	42	6.4 (3.1, 9.7)	31	6.2 (2.3, 10.1)	<0.01^1^	0.01v	0.32
3-methoxybenzoic acid	44	3.5 (1.0, 6.0)	46	3.9 (1.4, 6.3)	42	3.3 (0.71, 5.8)	31	4.7 (1.7, 7.6)	0.69	0.90	0.54
hippuric acid	44	11.8 (8.3, 15.3)	46	12.7 (9.3, 16.1)	42	7.6 (4.0, 11.2)	31	8.5 (4.4, 12.7)	0.07	0.02^1^	0.49

Values are mean (95% CI). Metabolites shown are those with a significant group, age or sex effect; *P* values calculated from linear regression.

1 False discovery rate adjusted *P* values < 0.2. Full dataset providing P values for all analytes is found in Supplement Table S6.

2 Metabolite isomers which could not be resolved effectively by HPLC were quantified according to one of the structural isomers.

### Group prediction

In linear discriminant analysis, 77% of younger participants and 62% of older participants were classified into the correct age groups based on their scores on the discriminant dimensions (Table 5). Hydroxy-methoxybenzoic acid, 2-hydroxycinnamic acid, 4-methoxybenzaldehyde (positive correlations), benzoic acid-4-*O*-glucuronide, and 3-methylhippuric acid (negative correlations) were the metabolites with the largest β-coefficients and therefore most likely to classify participants in the correct age group.

**TABLE 5:** Classification tables obtained from linear discriminant analysis in 163 participants from the COB study

Metabolite	Coefficient^1^	*P*
hydroxy-methoxybenzoic acid^2^	0.43	0.08
3-methylhippuric acid	−0.44	0.03
4-methoxybenzaldehyde	0.28	0.02
2-hydroxycinnamic acid	0.36	0.06
3-hydroxy-4-methoxybenzoic acid	0.17	0.01
3-(4-hydroxy-3-methoxyphenyl)propionic acid	0.27	0.13
4-hydroxy-3-methoxyacetophenone	0.23	0.17
3-(phenyl)propionic acid	0.18	0.08
3-(dihydroxyphenyl)propionic acid^2^	0.15	0.22
benzoic acid-4-*O*-glucuronide	−0.50	0.13

*P* = calculated using ANOVA.

1 Standardized canonical discriminant function coefficients representing the effect of a 1 SD increase on metabolite concentration on the SD increase in the predicted values on the discriminant function. These values indicate the predictive ability of these metabolites to classify participants into the correct age group.

2 Metabolites without numbered nomenclature are isomers of unknown hydroxy, methoxy, sulfate or glucuronide structural orientation (no physical references standards were available for retention time conformation but were matched for mass and MS/MS spectra).

### Gut microbiome composition by age and sex

Five taxa at the Family level—*Bacteroidaceae, Christensenellaceae, Clostridiales (unclassified), Dehalobacteriaceae,* and *Rikenellaceae*—displayed significant differences by age group (Q < 0.20, Supplementary Table S8). Relative abundance of all taxa was higher in younger participants, with the exception of *Bacteroidaceae,* which was higher in older participants. Relative abundance of *Barnesiellaceae* differed according to sex, with higher abundance in males. There were no significant differences in alpha diversity (Shannon Index) between older and younger individuals (*P* = 0.12) or males and females (*P* = 0.84) (data not shown). Hierarchical regression analysis revealed that the addition of sex and microbial composition to the model did not improve the prediction of metabolite concentrations over age (Table 6)

**TABLE 6:** Hierarchal regression analysis of predictors of metabolite concentrations in COB participants

Metabolite	*n*	M1	M2	M3
		β (95% CI) for age	*P*	*P*
hydroxy-methoxybenzoic acid^1^	156	0.04 (0.01, 0.07)	0.06	0.67
3-methylhippuric acid (x 10^2^)	155	−0.42 (−0.84, 0.00)	0.05	0.74
4-methoxybenzaldehyde	154	0.00 (0.05, 0.01)	0.94	0.97
2-hydroxycinnamic acid (x 10^2^)	156	0.02 (0.00, 0.05)	0.10	0.58
3-hydroxy-4-methoxybenzoic acid	155	0.06 (0.02, 0.09)	0.69	0.56
3-(4-hydroxy-3-methoxyphenyl)propionic acid	156	0.04 (−0.15, 0.08)	0.36	0.73
4-hydroxy-3-methoxyacetophenone	155	0.01 (−0.13, 0.02)	0.36	0.55
3-(phenyl)propionic acid	125	0.05 (−0.66, 0.11)	0.64	0.94
3-(dihydroxyphenyl)propionic acid^1^	154	0.01 (−0.75, 0.02)	0.39	0.58
benzoic acid-4-*O*-glucuronide(x 10^2^)	155	−0.74 (−1.62, 0.00)	0.06	0.43

M1 = age; M2 = age and sex; M3 = age and PCA1.

β (95% CI) for age were calculated from hierarchical regression with metabolite concentration as the dependent variable and age, sex and PCA1 as predictors. P= P value of the F-statistic indicating if the subsequent model offered any significant improvement over M1. M= model; PCA1 = a linear combination of the gut microbiome variables associated with age (first principal component; 41% of the variance).

1 Metabolite isomers which could not be resolved effectively by HPLC were quantified according to one of the structural isomers.

## Discussion

Overall, we observed no impact of age or sex on total cumulative 24-h urinary elimination of (poly)phenols. This lack of effect of age is consistent with a smaller previous study (*n* = 40), which looked at flavan-3-ol metabolism in men only and observed no difference in total “structurally related epicatchin metabolites” in plasma 0–6 h or urine 0–24 h. However, we did observe that overall age was the main determinant of flavonoid metabolite excretion kinetics over 24 h. Interestingly, adjustment for BMI and the gut microbial taxa had little effect on the significance of the observed relationships, indicating that any age-associated effects were not mediated by differences in gut microbial speciation or BMI. Older participants consumed considerably more flavonoids in their habitual diet, with nearly 3 times higher intakes compared with younger individuals, and with the highest intakes reported in older females; however, adjusting for habitual intake only moderately reduced the number of metabolites showing age effects (Supplemental Table 5).

The Cmax for individual microbial metabolites of (poly)phenols differed by either age or sex for 9 metabolites; however, there were no overall differences in cumulative excretion or Cmax for all polyphenol metabolites combined (i.e., total collective of (poly)phenol metabolites). Differences in Tmax were more pronounced for many metabolites, including both precursor (poly)phenols (hesperetin, naringenin, epicatechin, catechin, and cyanidin glucoside) and their microbial metabolites.

Metabolites displaying significant group, age, or sex effects for Tmax included hydroxybenzoic acids, 3-(phenyl)propionic acids, 5-(Hydroxyphenyl)-gamma-valerolactones and hippuric acids. Hydroxybenzoic acids, 3-(phenyl)propionic acids, and hippuric acids are reported to be derived from multiple (poly)phenol-rich food sources; whereas the 5-(hydroxyphenyl)-gamma-valerolactones were most likely derived from cocoa flavan-3-ols and procyanidins in cocoa and blackcurrant, hesperetin-3'-*O*-glucuronide from orange, and ellagic acid from blackcurrant [2,4,21,36]. Interestingly, there were no clear patterns in elimination kinetics (Cmax or Tmax) for precursor flavonoids relative to their conjugated metabolites (i.e., methyl, sulfate, and glucuronide conjugation) between age or sex groups.

A substantial fraction of flavonoid intake is subjected to gut microbial metabolism, structurally altering the precursor flavonoids found in the diet, forming smaller molecules such as phenolic and aromatic acids, which are more bioavailable and may be highly bioactive [1,4]. Although flavonoid intake has been shown to influence microbiota speciation [19,20], the role of microbiota functional and metabolic diversity in flavonoid bioavailability is poorly understood. In the present investigation, 5 taxa at the Family level displayed age differences; however, hierarchical regression revealed no effects of microbial composition or sex on metabolite concentration. Within our microbial composition dataset, alpha diversity was not significantly higher in older compared to younger individuals; however, other studies have presented a mixed picture of the association of aging with gut and microbial alpha diversity [49].

Tmax differed by as much as 3–5 h between the age groups and was typically later in older individuals. This finding highlights the need for future studies to consider longer sampling strategies when recruiting wide age ranges for exploring metabolite bioactivity and attempting to establish correlations between peak blood concentration of flavonoid metabolites and health/disease status biomarkers (e.g., glucose or lipoprotein homeostasis). The etiology of differences in Tmax between older and younger individuals is unknown and was not explained by differences in BMI or microbiome speciation. They may derive from differences in glomerular filtration rates, liver metabolism, or gastric and intestinal transit time [50,51] affecting food digestibility and absorption and elimination of microbial metabolites, variables that should be captured where possible in future studies.

Precursor flavonoids from citrus, cocoa, and berries (naringenin, epicatechin, and cyanidin) and their previously reported metabolites, including microbial metabolites (hippuric acids, benzoic acids, and valerolactones) [2,4,21,36] were graphically depicted (Supplementary Figures 1-13) to visualize if differences in rates of elimination of precursor flavonoids across age or sex groups differed or were predictive of their metabolite elimination. Rates of elimination (i.e., slopes of cumulative elimination curves) appeared consistent across age and sex, with older individuals generally having higher Cmax and later Tmax than younger individuals for these perceived biomarkers of flavonoid intake. This evidence indicates that monitoring a limited number of perceived intake biomarkers in nutrition interventions is likely to poorly reflect actual shifts in the dietary metabolomes of individuals and highlights the importance of using more global metabolomics approaches in future interventions.

In the present study, both standard (*P* < 0.05) and conservative statistical approaches (Q < 0.20; Benjamini-Hochberg method for false discovery) were utilized to emphasize the potential impact of multiple testing when characterizing large numbers of metabolites. Moving forward, polyphenol interventions using untargeted or quantitative metabolomics approaches will require consideration and consensus on the most appropriate statistical practices.

The present study design was relatively unique for flavonoid (and (poly)phenols in general) interventions as participants were prospectively recruited by age and sex, allowing for the first comprehensive investigation of the impact of sex, age, and microbiota speciation on acute flavonoid absorption, metabolism, and elimination following the consumption of a flavonoid enriched test meal. The elimination of flavonoids from the background diet 48 h prior to the intervention was an additional strength as it standardized and minimized the contribution of background diet derived metabolites detected in urine post-test meal. The present study was, however, unable to pinpoint a possible mechanism behind the variability in elimination. The lack of characterization of additional possible mediators, such as gastric emptying, intestinal transit, liver and kidney function (glomerular filtration rate), and genetic polymorphisms in phase 1 and 2 metabolism, is identified as a study limitation, along with a lack of capture of metabolite concentrations beyond 24 h, which would have resulted in gut-derived metabolites not being fully recovered. However, it was decided during the trial design stage to limit further participant burden caused by the restricted diet and repeated sampling over an additional 24 h. Many phytochemical phase II metabolites are/were not commercially available as reference standards for use in quantitative analysis (e.g., glucuronide or sulfate conjugates of flavan-3-ols, valerolactones, phenylvaleric acids, etc.), and therefore, the present total recovery is likely to be an underestimation of the amount and diversity of polyphenol metabolites excreted. Further, there is likely to be some minimal contribution of endogenous and dietary aromatic amino acids to the phenolic acid metabolite pools quantified in the urine in the present study, such as hippuric acids; however, all participants were on the same intervention meals, and the exclusion diet was essentially void of (poly)phenols, minimizing this possible confounding of endogenous or dietary substrates. Finally, the research was conducted in a healthy population, with > 90% of our study population being White British, and extrapolation of findings to clinical groups with significant disease pathology and medication use or other ethnic groups should be done with caution.

In conclusion, our study provides evidence of a large impact of age on the elimination kinetics of (poly)phenol metabolites, particularly Tmax, which occurs much later in older individuals. The age effects observed on individual metabolite recovery do not appear to be substantially driven by differences in background diet, BMI, and microbiota speciation. Our results indicate that evaluating blood or urine signatures of (poly)phenol metabolites at a single time point is unlikely to capture the true absorption and elimination kinetics across age and sex groups.

## Author contributions

The authors’ responsibilities were as follows – AMM, AC: designed the study; SH, NT, BCD, DB, SL: were responsible for participant recruitment, delivery of the intervention protocol, and the collection, processing, and storage of biological samples; NT, BCD, CDK: designed, delivered, and interpreted the urinary flavonoid analysis; LCC, DM: were responsible for the preparation of the fecal samples for microbiome speciation and the interpretation of the data; AJ: conducted all statistical analyses; CDK, AJ, AMM: drafted the paper; all authors: read and approved the final manuscript.

## Data availability

Data described in the manuscript, including urine concentration, analytical methodologies, and statistical analysis data files, will be made available upon request and approval from the corresponding author.

## Funding

This work was funded by a Biotechnology and Biological Sciences Research Council (BBSRC), Food and Health, Institute Strategic Programme Grant (ISP) (BB/J004545/1), who had no role in the design or delivery of the study or in the data analysis and its interpretation.

## Conflict of interest

AMM reports was provided by University of East Anglia Norwich Medical School. If there are other authors, they declare that they have no known competing financial interests or personal relationships that could have appeared to influence the work reported in this paper.
