# Supplementary material for: Effect of age and sex on the urinary elimination of a single dose of mixed flavonoids: results from a single-arm intervention in healthy United Kingdom adults
Source: Am J Clin Nutr. 2025 May 12;122(1):101–11. doi: 10.1016/j.ajcnut.2025.05.006 (PMC12489340; doi:10.1016/j.ajcnut.2025.05.006)
Supplement: Multimedia component 2 [file mmc2.pptx]

## Slide 1
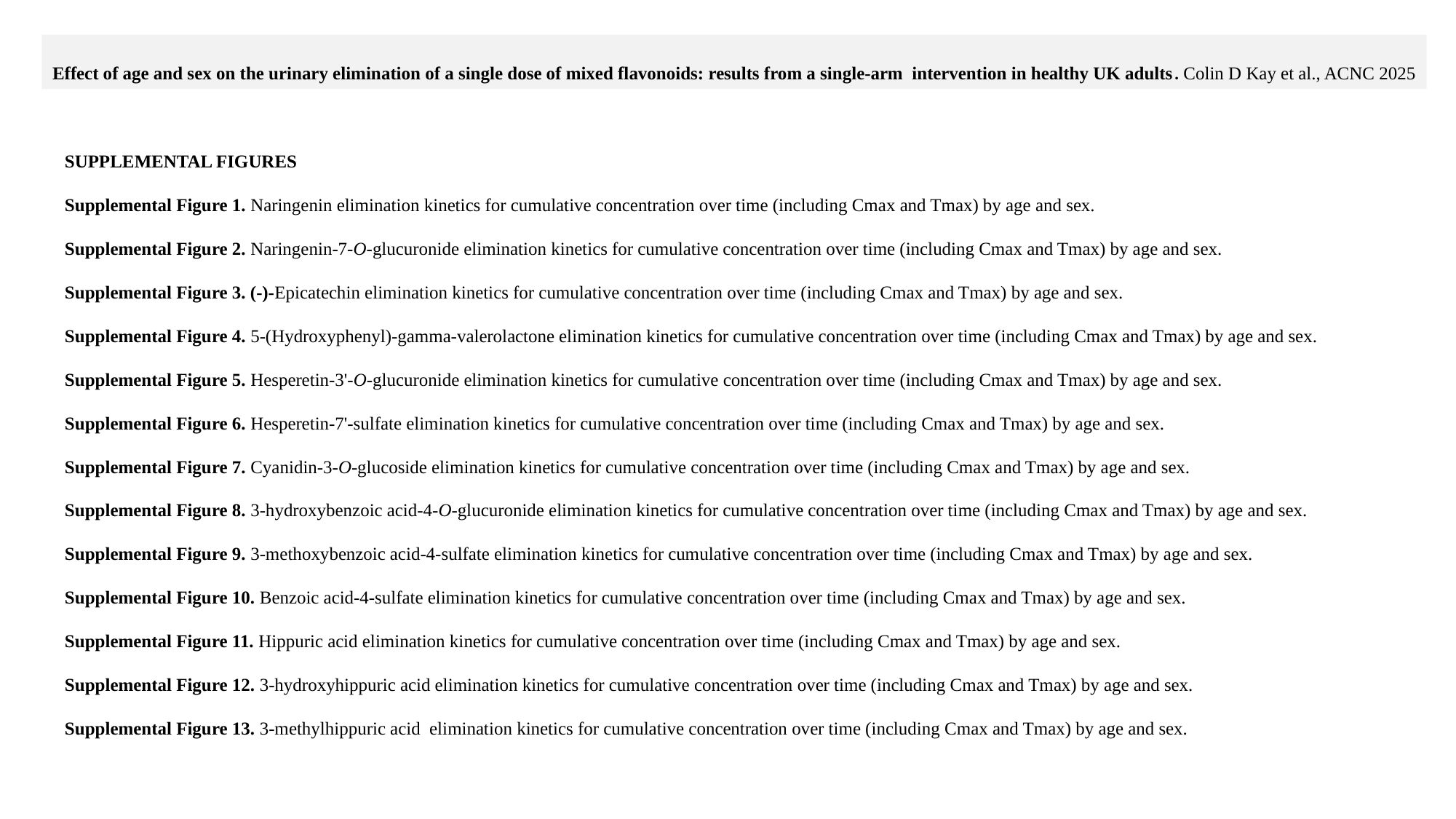

Effect of age and sex on the urinary elimination of a single dose of mixed flavonoids: results from a single-arm intervention in healthy UK adults. Colin D Kay et al., ACNC 2025
SUPPLEMENTAL FIGURES
Supplemental Figure 1. Naringenin elimination kinetics for cumulative concentration over time (including Cmax and Tmax) by age and sex.
Supplemental Figure 2. Naringenin-7-O-glucuronide elimination kinetics for cumulative concentration over time (including Cmax and Tmax) by age and sex.
Supplemental Figure 3. (-)-Epicatechin elimination kinetics for cumulative concentration over time (including Cmax and Tmax) by age and sex.
Supplemental Figure 4. 5-(Hydroxyphenyl)-gamma-valerolactone elimination kinetics for cumulative concentration over time (including Cmax and Tmax) by age and sex.
Supplemental Figure 5. Hesperetin-3'-O-glucuronide elimination kinetics for cumulative concentration over time (including Cmax and Tmax) by age and sex.
Supplemental Figure 6. Hesperetin-7'-sulfate elimination kinetics for cumulative concentration over time (including Cmax and Tmax) by age and sex.
Supplemental Figure 7. Cyanidin-3-O-glucoside elimination kinetics for cumulative concentration over time (including Cmax and Tmax) by age and sex.
Supplemental Figure 8. 3-hydroxybenzoic acid-4-O-glucuronide elimination kinetics for cumulative concentration over time (including Cmax and Tmax) by age and sex.
Supplemental Figure 9. 3-methoxybenzoic acid-4-sulfate elimination kinetics for cumulative concentration over time (including Cmax and Tmax) by age and sex.
Supplemental Figure 10. Benzoic acid-4-sulfate elimination kinetics for cumulative concentration over time (including Cmax and Tmax) by age and sex.
Supplemental Figure 11. Hippuric acid elimination kinetics for cumulative concentration over time (including Cmax and Tmax) by age and sex.
Supplemental Figure 12. 3-hydroxyhippuric acid elimination kinetics for cumulative concentration over time (including Cmax and Tmax) by age and sex.
Supplemental Figure 13. 3-methylhippuric acid  elimination kinetics for cumulative concentration over time (including Cmax and Tmax) by age and sex.

## Slide 2
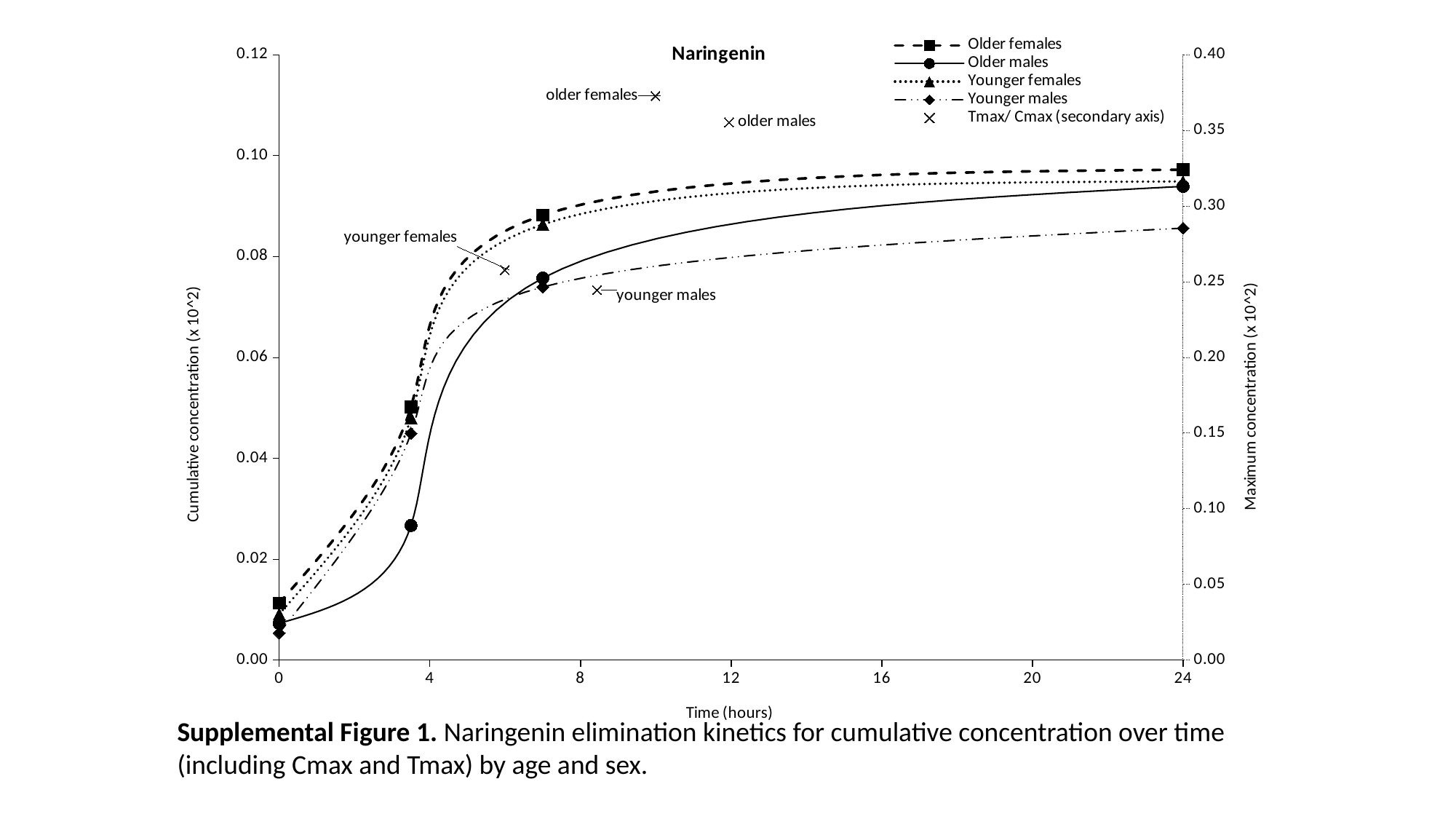

### Chart: Naringenin
| Category | Older females | Older males | Younger females | Younger males | Tmax/ Cmax (secondary axis) |
|---|---|---|---|---|---|Supplemental Figure 1. Naringenin elimination kinetics for cumulative concentration over time (including Cmax and Tmax) by age and sex.

## Slide 3
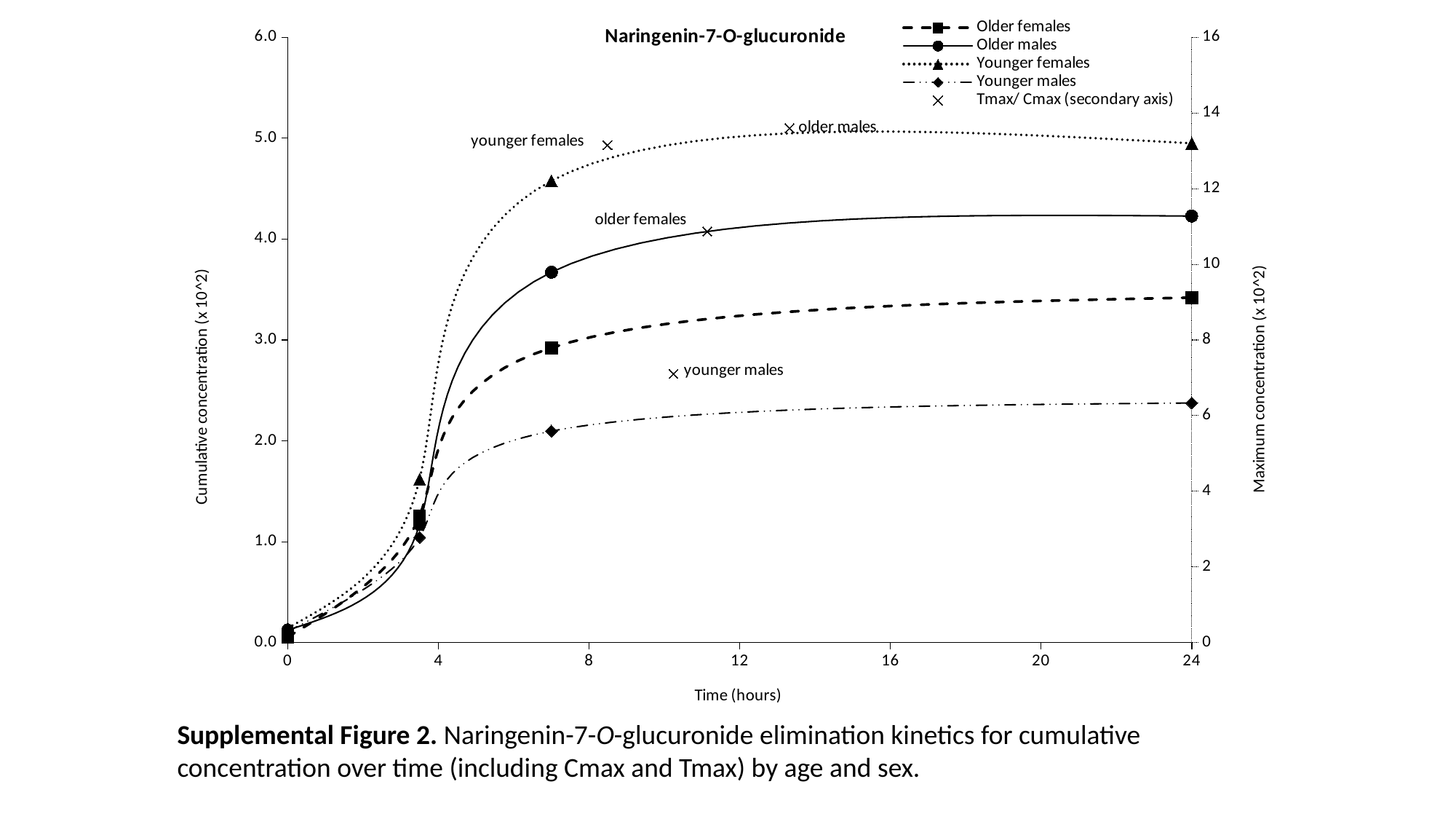

### Chart: Naringenin-7-O-glucuronide
| Category | Older females | Older males | Younger females | Younger males | Tmax/ Cmax (secondary axis) |
|---|---|---|---|---|---|Supplemental Figure 2. Naringenin-7-O-glucuronide elimination kinetics for cumulative concentration over time (including Cmax and Tmax) by age and sex.

## Slide 4
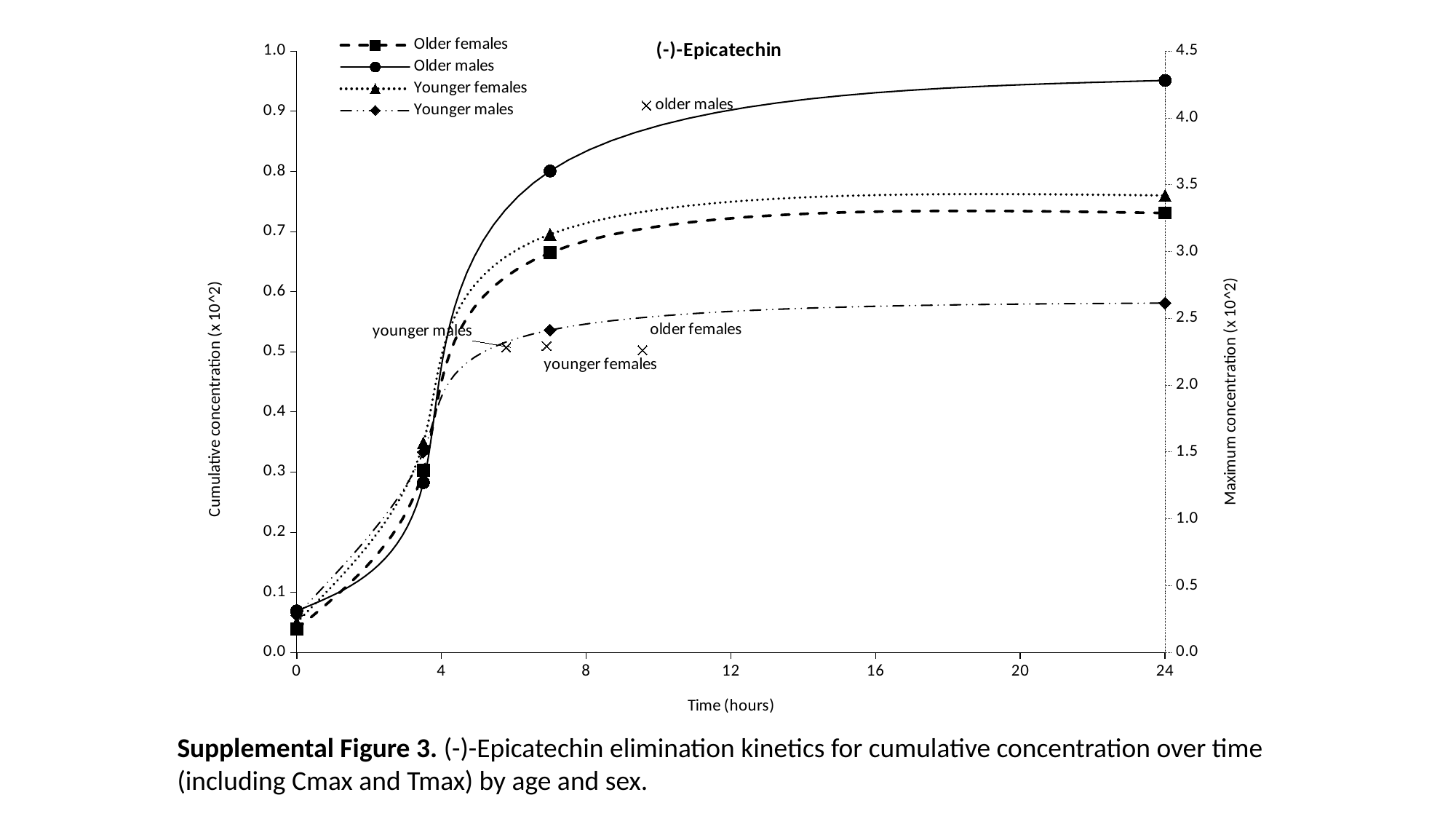

### Chart: (-)-Epicatechin
| Category | Older females | Older males | Younger females | Younger males | Tmax/ Cmax (secondary axis) |
|---|---|---|---|---|---|Supplemental Figure 3. (-)-Epicatechin elimination kinetics for cumulative concentration over time (including Cmax and Tmax) by age and sex.

## Slide 5
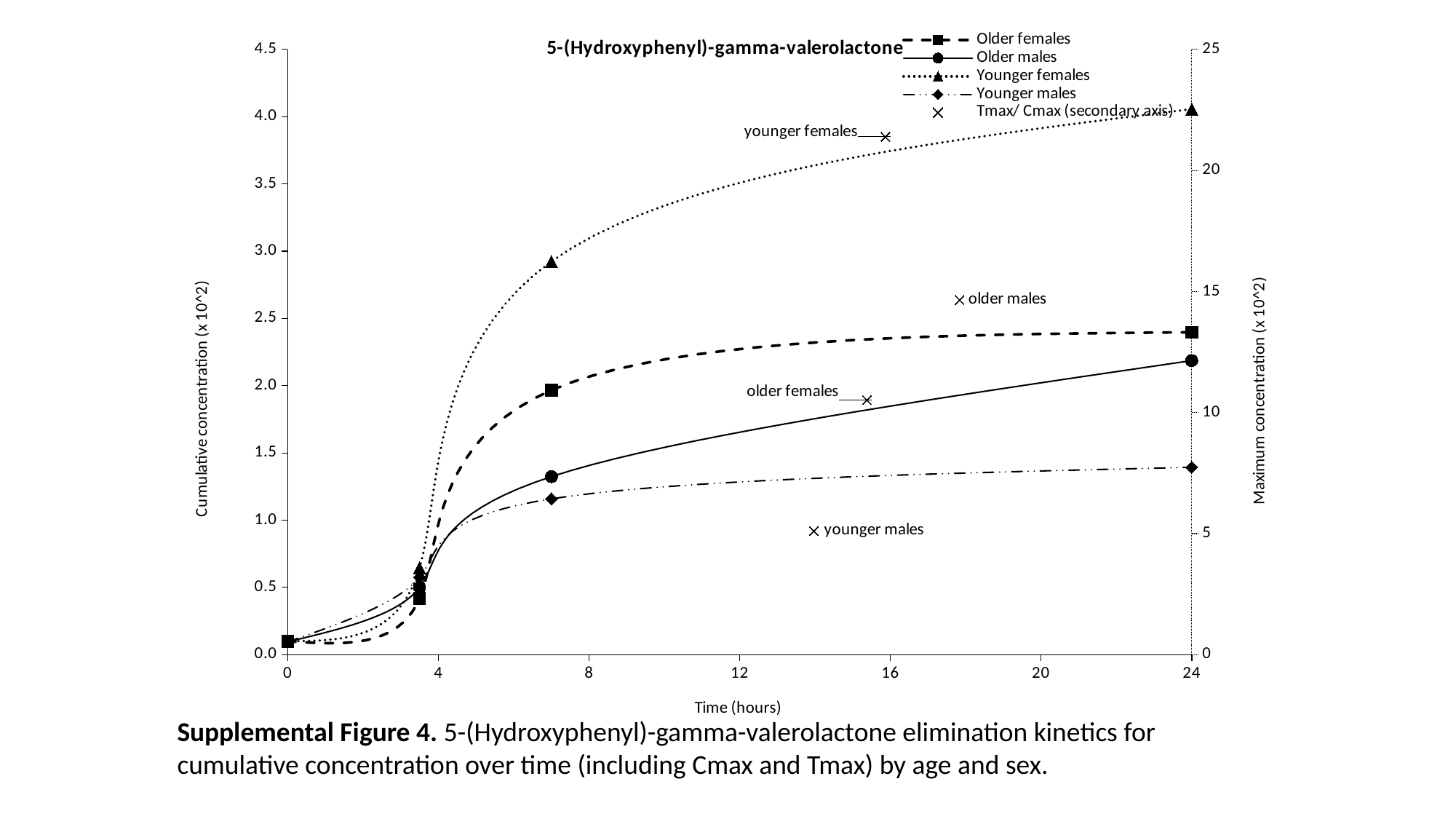

### Chart: 5-(Hydroxyphenyl)-gamma-valerolactone
| Category | Older females | Older males | Younger females | Younger males | Tmax/ Cmax (secondary axis) |
|---|---|---|---|---|---|Supplemental Figure 4. 5-(Hydroxyphenyl)-gamma-valerolactone elimination kinetics for cumulative concentration over time (including Cmax and Tmax) by age and sex.

## Slide 6
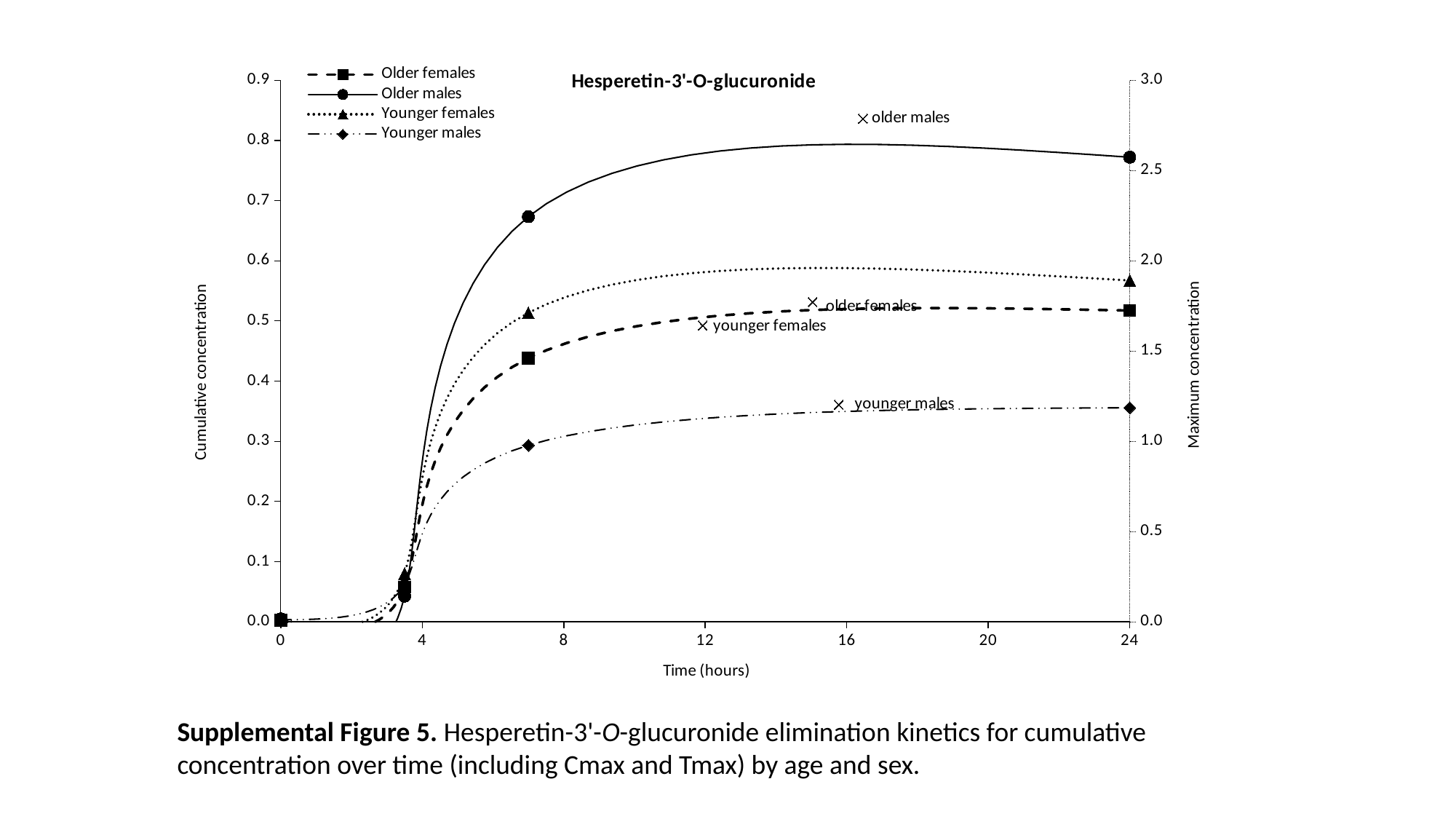

### Chart: Hesperetin-3'-O-glucuronide
| Category | Older females | Older males | Younger females | Younger males | Tmax/ Cmax (secondary axis) |
|---|---|---|---|---|---|Supplemental Figure 5. Hesperetin-3'-O-glucuronide elimination kinetics for cumulative concentration over time (including Cmax and Tmax) by age and sex.

## Slide 7
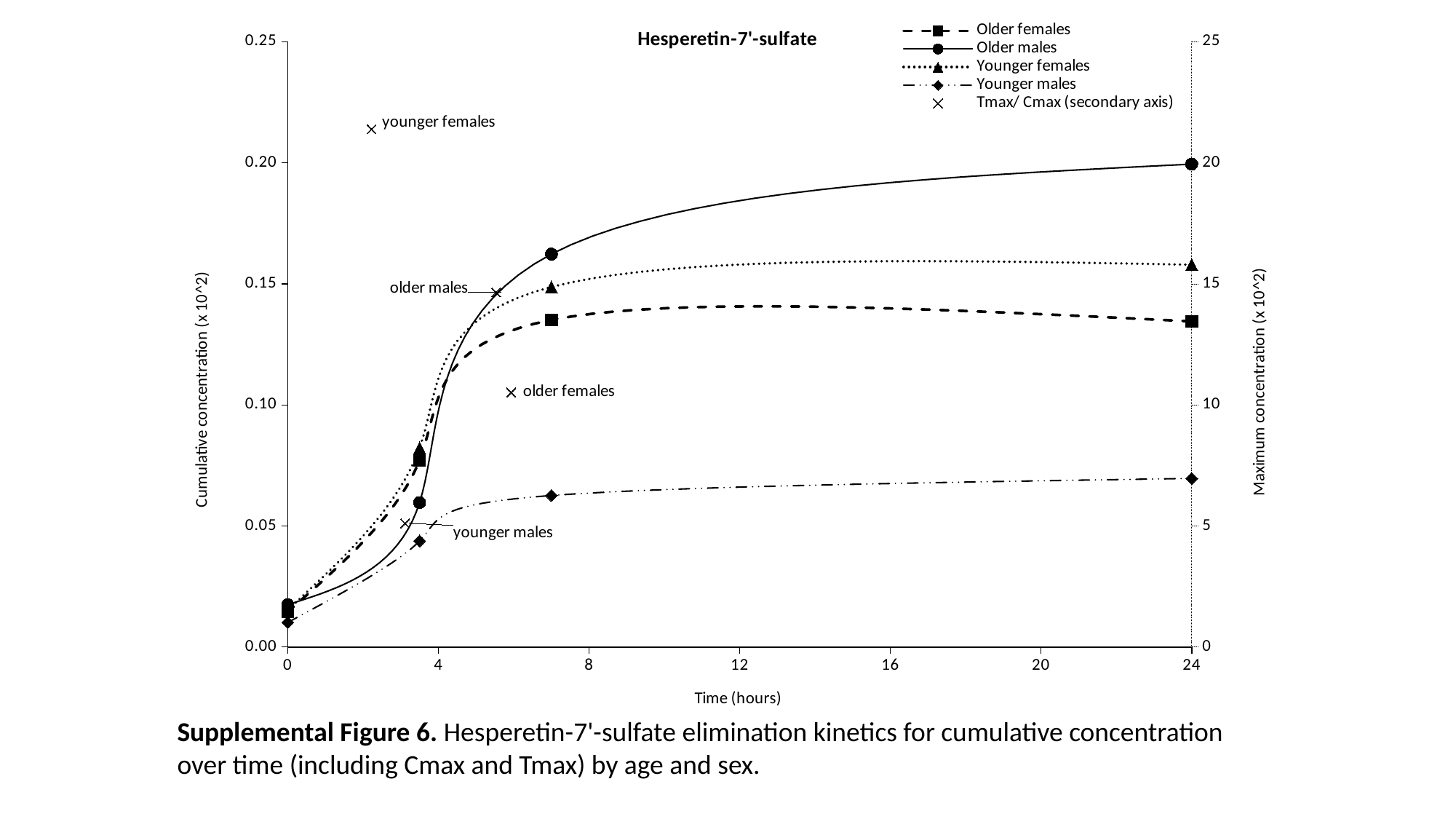

### Chart: Hesperetin-7'-sulfate
| Category | Older females | Older males | Younger females | Younger males | Tmax/ Cmax (secondary axis) |
|---|---|---|---|---|---|Supplemental Figure 6. Hesperetin-7'-sulfate elimination kinetics for cumulative concentration over time (including Cmax and Tmax) by age and sex.

## Slide 8
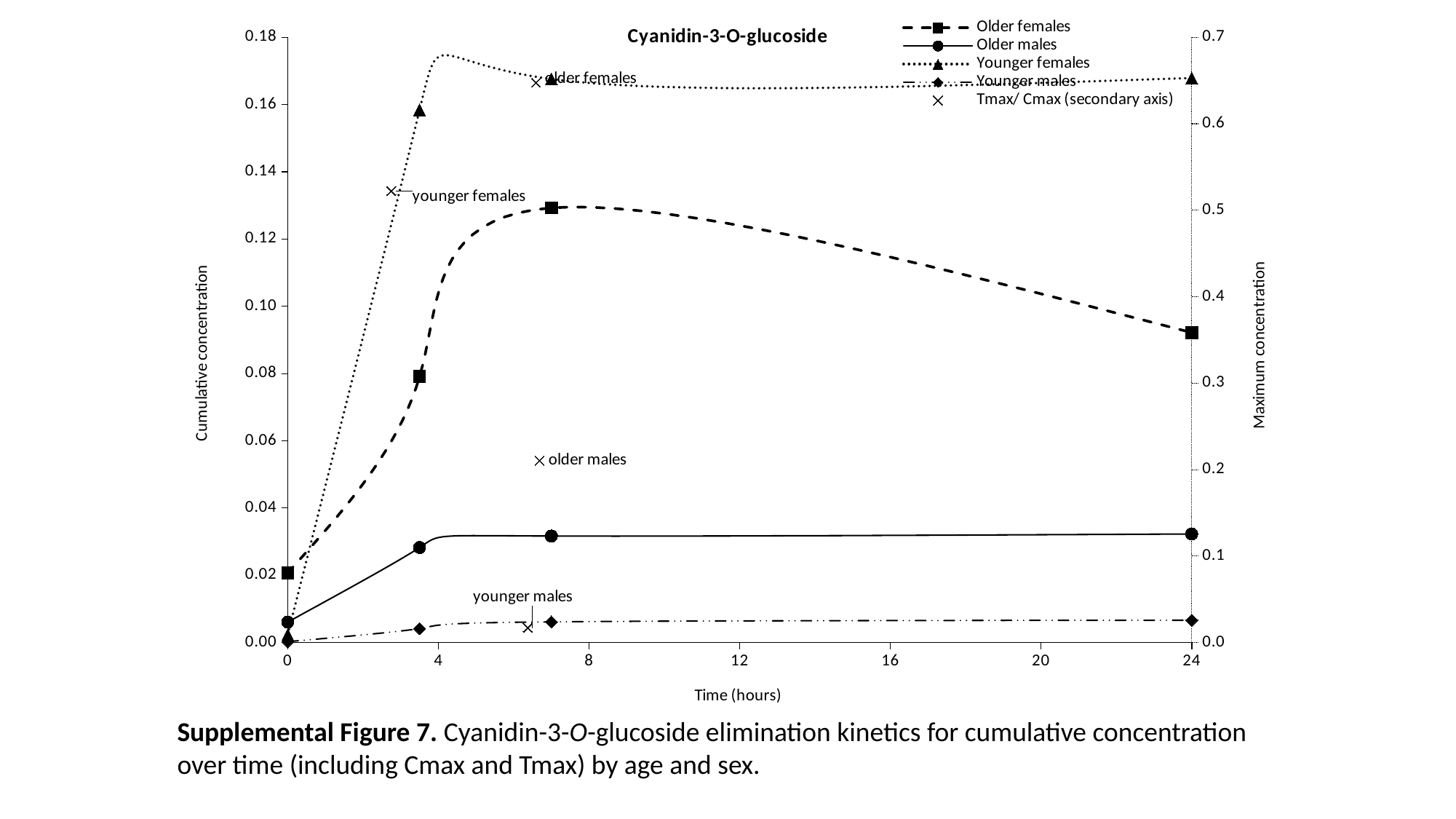

### Chart: Cyanidin-3-O-glucoside
| Category | Older females | Older males | Younger females | Younger males | Tmax/ Cmax (secondary axis) |
|---|---|---|---|---|---|Supplemental Figure 7. Cyanidin-3-O-glucoside elimination kinetics for cumulative concentration over time (including Cmax and Tmax) by age and sex.

## Slide 9
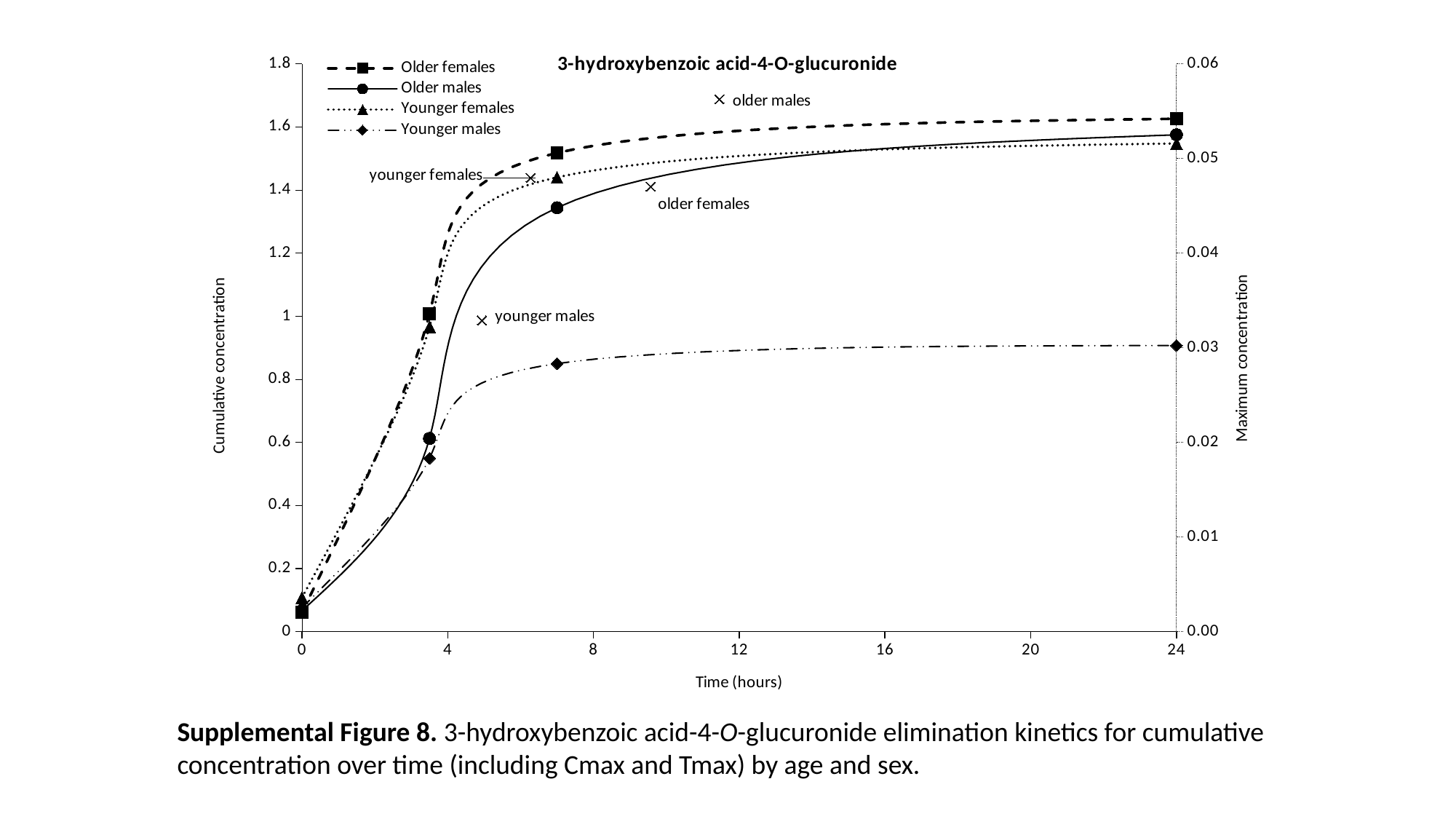

### Chart: 3-hydroxybenzoic acid-4-O-glucuronide
| Category | Older females | Older males | Younger females | Younger males | Tmax/ Cmax (secondary axis) |
|---|---|---|---|---|---|Supplemental Figure 8. 3-hydroxybenzoic acid-4-O-glucuronide elimination kinetics for cumulative concentration over time (including Cmax and Tmax) by age and sex.

## Slide 10
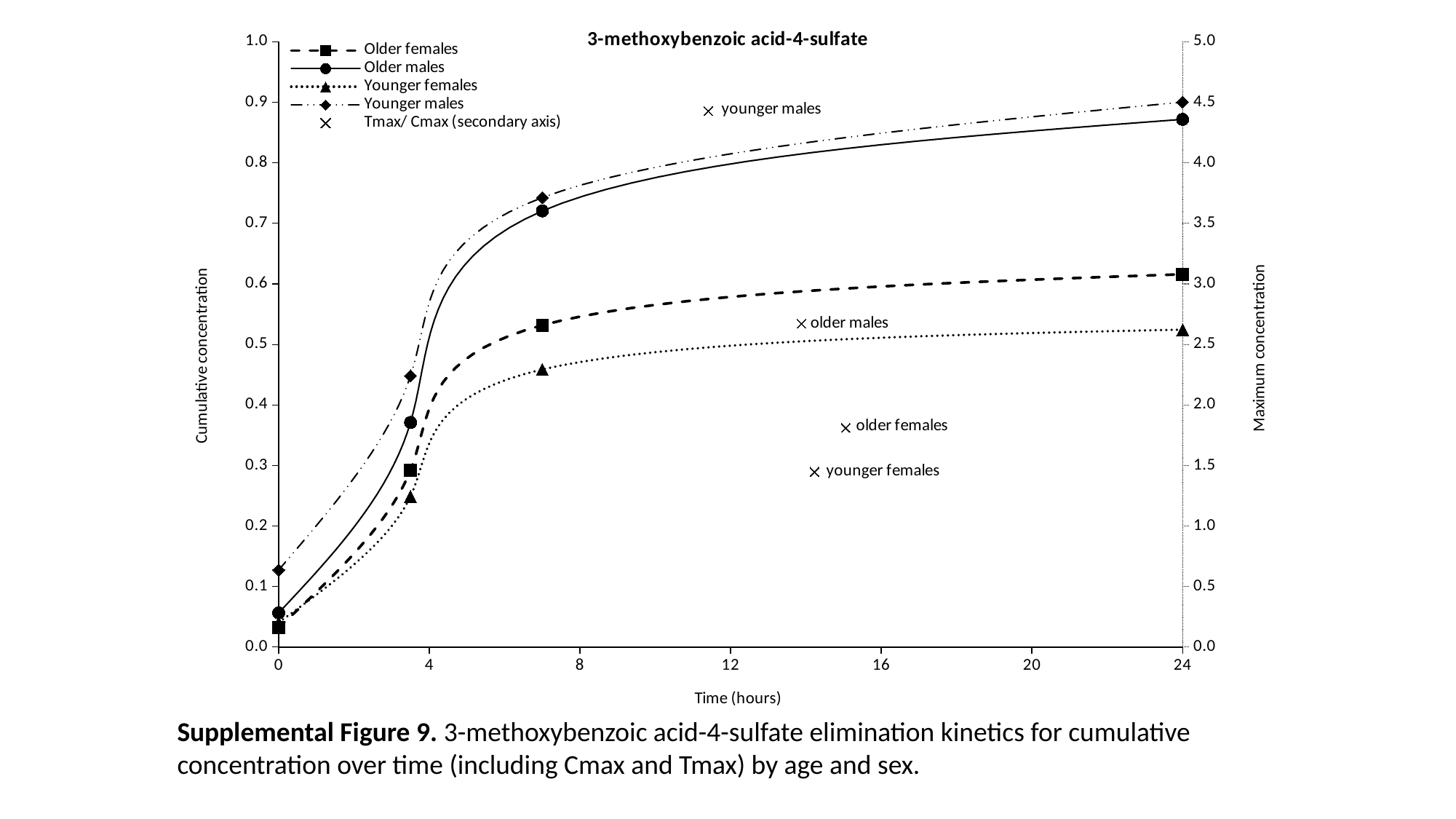

### Chart: 3-methoxybenzoic acid-4-sulfate
| Category | Older females | Older males | Younger females | Younger males | Tmax/ Cmax (secondary axis) |
|---|---|---|---|---|---|Supplemental Figure 9. 3-methoxybenzoic acid-4-sulfate elimination kinetics for cumulative concentration over time (including Cmax and Tmax) by age and sex.

## Slide 11
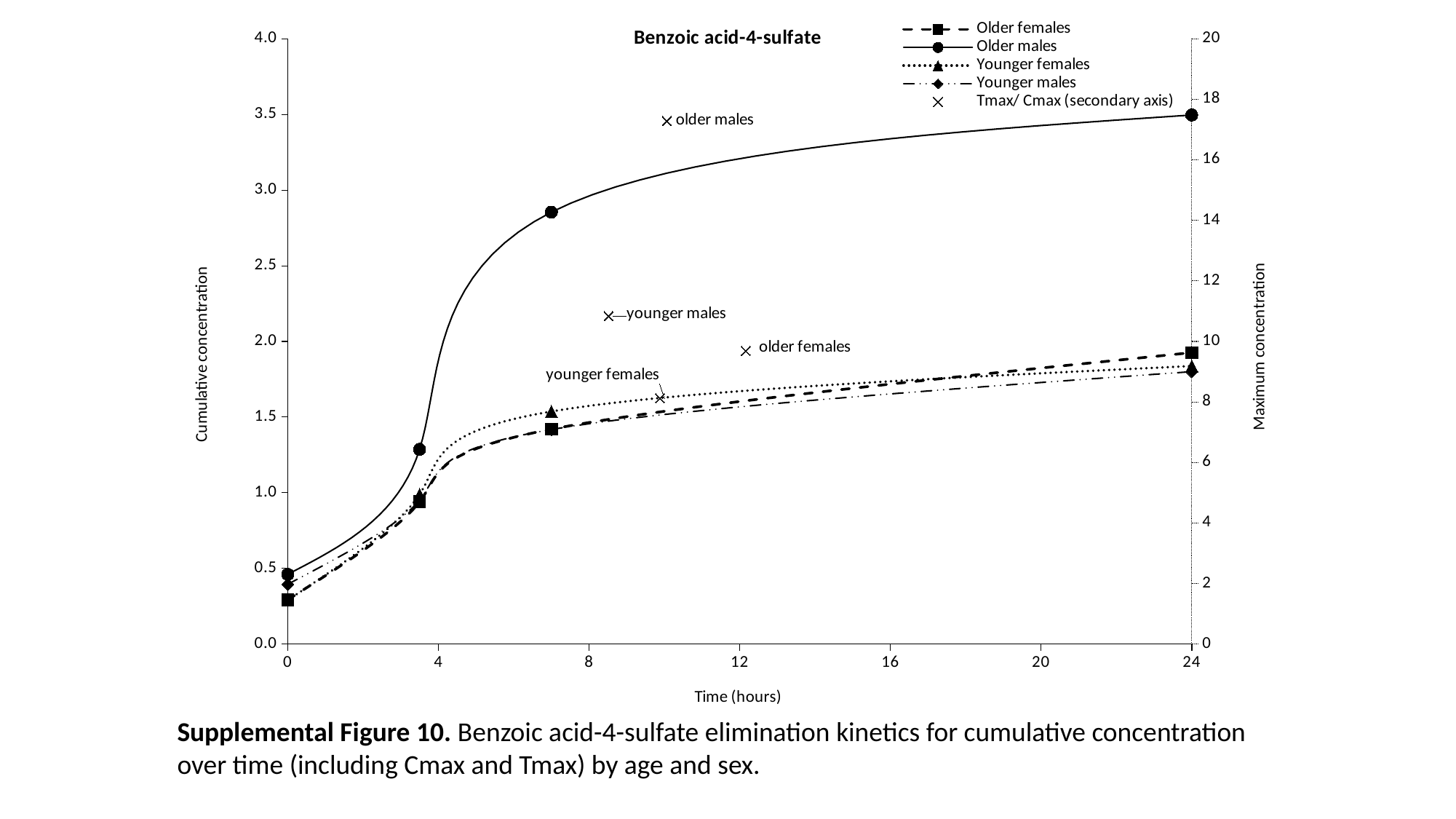

### Chart: Benzoic acid-4-sulfate
| Category | Older females | Older males | Younger females | Younger males | Tmax/ Cmax (secondary axis) |
|---|---|---|---|---|---|Supplemental Figure 10. Benzoic acid-4-sulfate elimination kinetics for cumulative concentration over time (including Cmax and Tmax) by age and sex.

## Slide 12
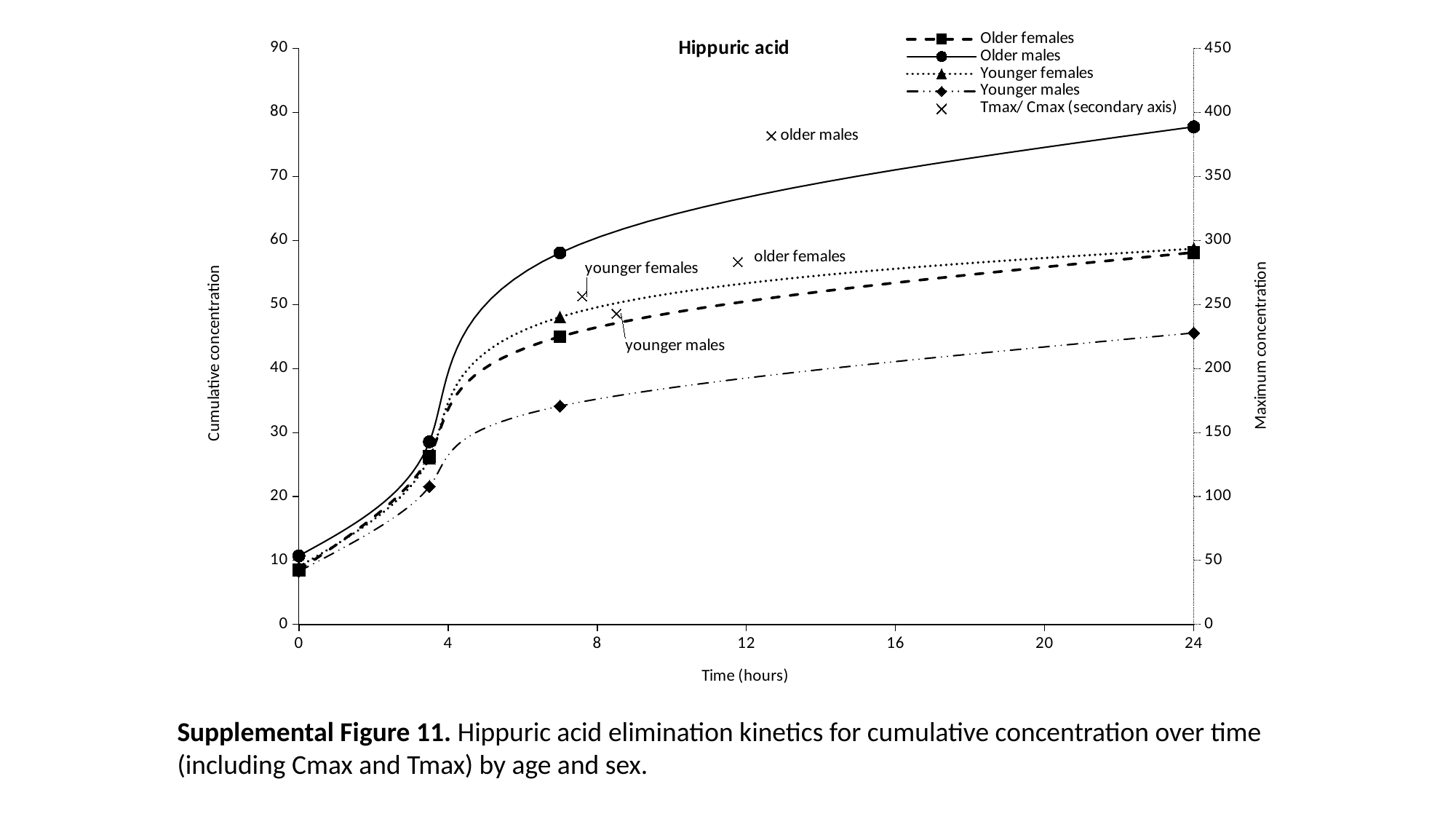

### Chart: Hippuric acid
| Category | Older females | Older males | Younger females | Younger males | Tmax/ Cmax (secondary axis) |
|---|---|---|---|---|---|Supplemental Figure 11. Hippuric acid elimination kinetics for cumulative concentration over time (including Cmax and Tmax) by age and sex.

## Slide 13
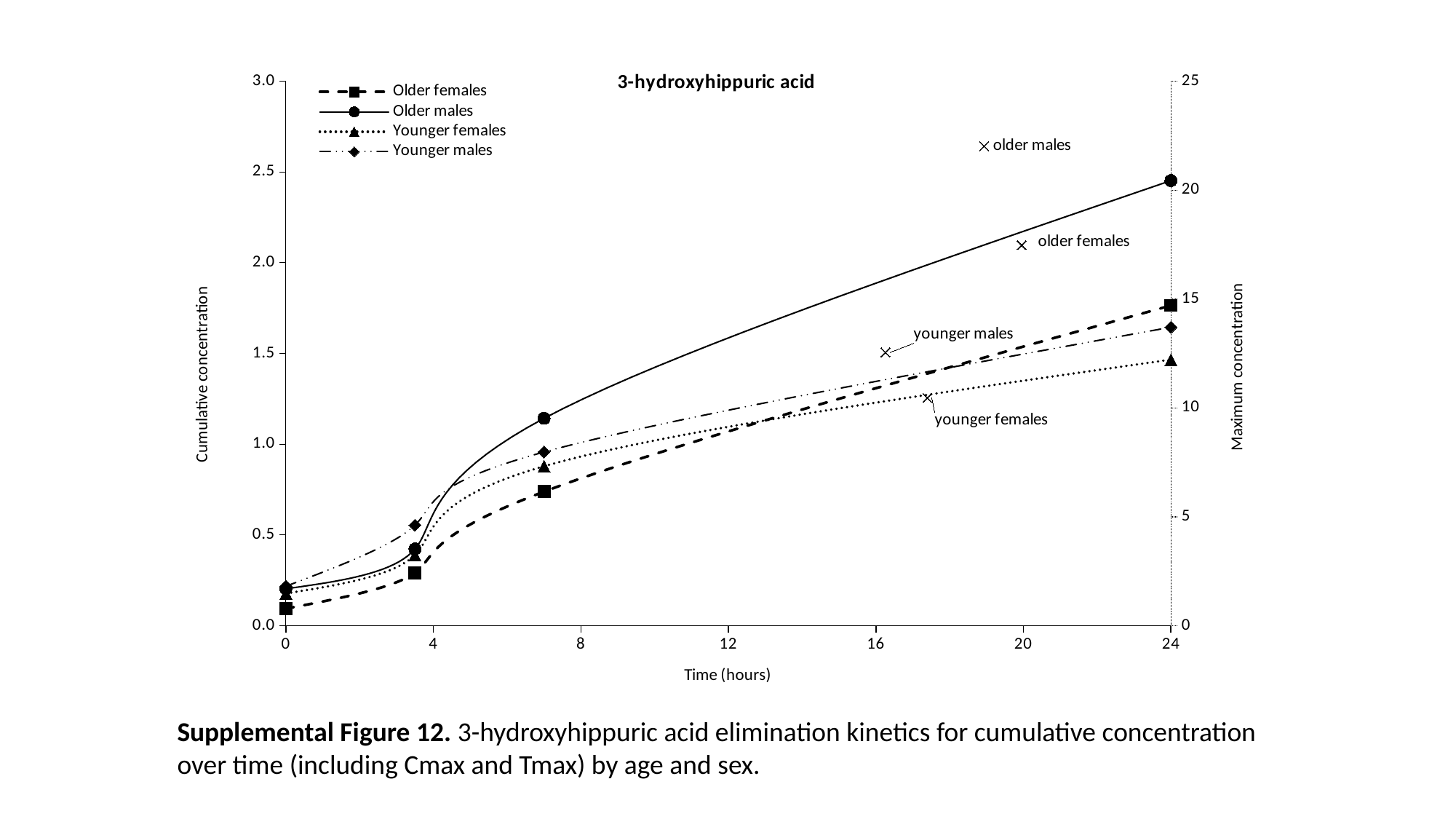

### Chart: 3-hydroxyhippuric acid
| Category | Older females | Older males | Younger females | Younger males | Tmax/ Cmax (secondary axis) |
|---|---|---|---|---|---|Supplemental Figure 12. 3-hydroxyhippuric acid elimination kinetics for cumulative concentration over time (including Cmax and Tmax) by age and sex.

## Slide 14
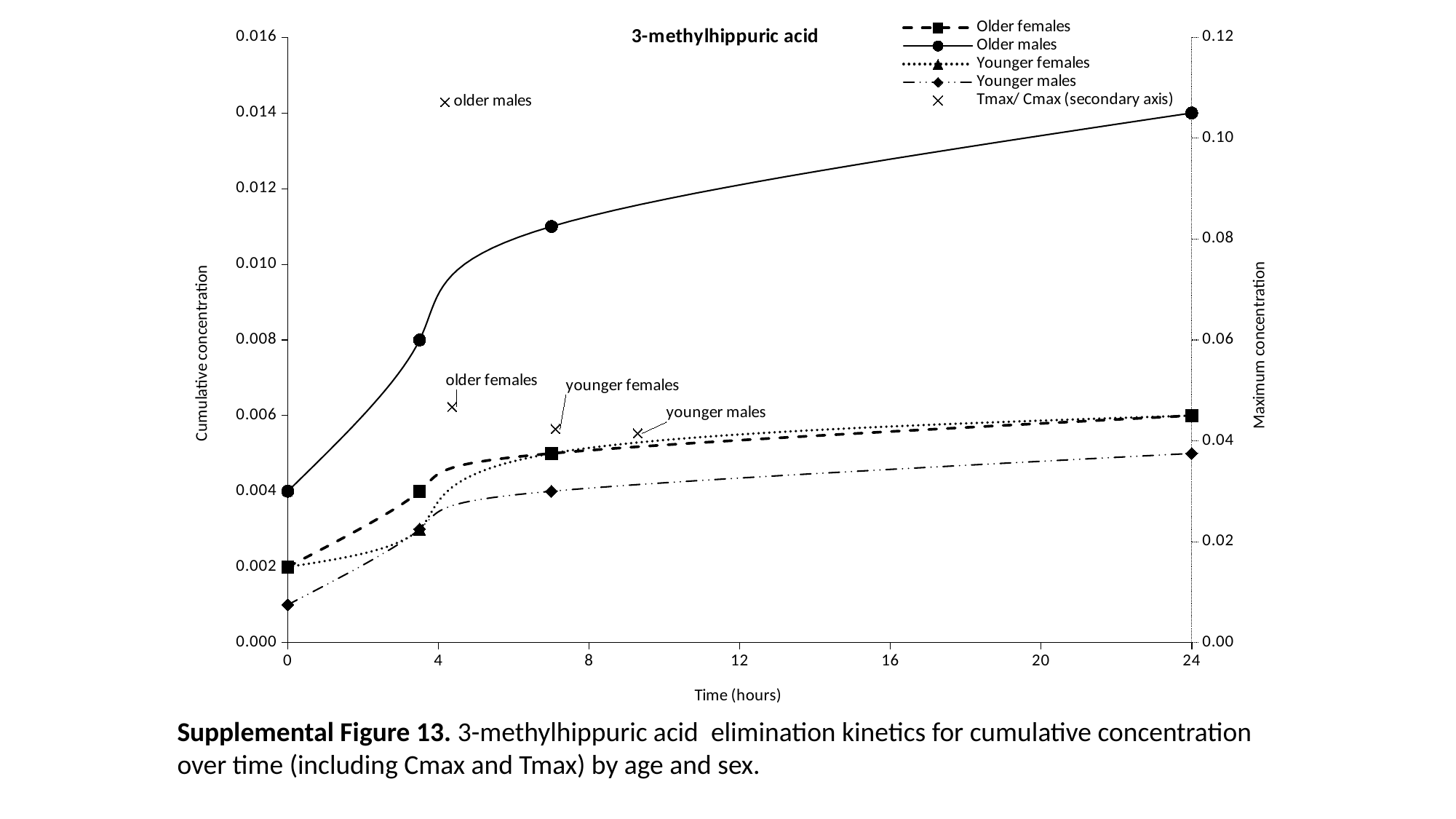

### Chart: 3-methylhippuric acid
| Category | Older females | Older males | Younger females | Younger males | Tmax/ Cmax (secondary axis) |
|---|---|---|---|---|---|Supplemental Figure 13. 3-methylhippuric acid elimination kinetics for cumulative concentration over time (including Cmax and Tmax) by age and sex.
